# Synthesis, Photoluminescence, and Electroluminescence of Phosphorescent Dipyrido[3,2-*a*;2′3′-*c*]phenazine–Platinum(II) Complexes Bearing Hole-Transporting Acetylide Ligands

**DOI:** 10.3390/molecules29163849

**Published:** 2024-08-14

**Authors:** Hiroki Matsuura, Naoki Okamura, Masaki Nagaoka, Naoya Suzuki, Shintaro Kodama, Takeshi Maeda, Shigeyuki Yagi

**Affiliations:** 1 Department of Applied Chemistry, Graduate School of Engineering, Osaka Prefecture University, 1-1 Gakuen-cho, Naka-ku, Sakai 599-8531, Osaka, Japannaoki_okamura@osakafu-u.net (N.O.); nsuzuki@omu.ac.jp (N.S.); skodama@omu.ac.jp (S.K.); tmaeda@omu.ac.jp (T.M.); 2 Department of Applied Chemistry, Graduate School of Engineering, Osaka Metropolitan University, 1-1 Gakuen-cho, Naka-ku, Sakai 599-8531, Osaka, Japan; su23437o@st.omu.ac.jp

**Keywords:** dipyrido[3,2-*a*;2′3′-*c*]phenazine, platinum complex, phosphorescence, organic light-emitting diode, non-doped device, solution-processing, multilayer device, orthogonal solvent system

## Abstract

In this study, novel phosphorescent dipyrido[3,2-*a*;2′3′-*c*]phenazine (dppz)–platinum(II)–phenylacetylide complexes were developed to fabricate non-doped organic light-emitting diodes (OLED) by solution-processing. To facilitate the charge carrier injection into the emitting layer (EML), 3,6-di-*tert*-butylcarbazole-functinalized phenylacetylides were employed. As for the dppz ligand, 9,9-dihexylfluoren-2-yl and 4-hexylthiophen-2-yl side-arms were introduced to the 2,7-positions, which led to reddish orange and red photoluminescence (PL), respectively, in solution and film states (PL wavelength: ca. 600 and ca. 625 nm, respectively). The carbazole-appended phenylacetylide ligands hardly affected the emission color, although unsubstituted phenylacetylides gave rise to aggregate- or excimer-based near-infrared PL with a low quantum yield. Two types of non-doped OLEDs were fabricated: single-layer and multilayer devices. In both devices, the organic layers were fabricated by spin-coating, and the EML consisted of a neat film of the corresponding platinum(II) complex. Therein, electroluminescence spectra corresponding to those of PL were observed. The single-layer devices exhibited low device efficiencies due to a deteriorated charge carrier balance. The multilayer devices possessed hole- and electron-transporting layers on the anode and cathode sides of the EML, respectively. Owing to an improved charge carrier balance, the multilayer devices exhibited higher device performance, affording considerably improved values of luminance and external quantum efficiency.

## 1. Introduction

Since the early-stage development of organic thin film-based electroluminescent devices by Tang’s group, organic light-emitting diodes (OLED) have been making great progress [1,2] and, nowadays, are applied to full-color flat panel displays such as TV sets and mobile phones, lighting apparatuses, and so on [3,4,5,6,7,8,9]. In the case of OLEDs, emitting materials are key constituents and affect the device efficiency to a considerable extent. Under an electric current, the emitter molecules are excited through the charge recombination of holes and electrons injected from the anode and the cathode, respectively, the excitons of which are generally divided into the singlet and triplet states in a ratio of 1:3 according to the spin-statistics theorem. Therefore, phosphorescent emitters have so far attracted considerable attention as the emitting materials for OLEDs because the combination of statistically generated triplet excitons (75%) with substantially generated triplet excitons (25%) via the singlet-to-triplet intersystem crossing (ISC) allows us to achieve the internal quantum efficiency (*η*_int_) of 100%, four times larger than the theoretical maximum value of *η*_int_ for traditional fluorescent emitters [10]. In this context, organometallic complexes with heavy metal centers, represented by platinum(II) [11,12,13,14,15,16,17] and iridium(III) [18,19,20,21,22,23,24] complexes, are good candidates for phosphorescent emitters, because strong spin–orbit coupling, resulting in the efficient mixing of excited singlet and triplet states via ISC, facilitates the generation of triplet excitons.

From the viewpoint of low-cost device fabrication for OLEDs, it has so far been discussed that solution-processing, including spin-coating and inkjet printing, is superior to vacuum deposition processing [25,26,27]. However, in the case of solution-processing, it is difficult to obtain multilayer device structures due to difficulties in finding orthogonal solvents to cast different layers. To overcome this problem, Pu and coworkers achieved multilayer OLEDs based on small molecules by carefully avoiding the interfacial disorder between neighboring organic layers [28]. OLEDs based on fluorescent conjugated polymers are good candidates as an emitting layer (EML) for a solution-processed device [29,30,31]. In this regard, phosphorescent polymer materials have been developed, where phosphorescent heavy metal complex units are attached to a polymer backbone as pendant phosphors or incorporated into the main chain of a conjugated polymer [32,33,34]. Phosphorescent organometallic complexes with high molecular weights, showing excellent film-forming properties, have also been developed to fabricate non-doped EMLs, as represented by platinum(II) and iridium(III) emitters bearing charge-carrier-transporting dendrons [35,36,37,38,39]. We recently reported sky-blue phosphorescent dendritic iridium(III) complexes, where charge-carrier-transporting dendrons are incorporated into an organoiridium(III) core [37,38]. The attached bulky dendrons effectively suppress aggregate formation even in the film state, although the film of a mixture of the separated dendron and the core complex exhibits aggregate-based green emission. These dendritic iridium(III) emitters are soluble in cyclohexane and insoluble in lower alcohols. Thus, the neat film is fabricated on a hole-transporting polymer such as poly(9-vinylcarbazole) (PVCz), using a cyclohexane ink solution, because PVCz is insoluble in cyclohexane. Furthermore, a neat film of an electron-transporting material such as [1,1′:3′,1″-terphenyl]-4,4″-diylbis(diphenylphosphine oxide) (BPOPB) is prepared by using a 2-propanol ink solution, without rinsing the iridium(III)-based film beneath. As a result, a multi-stacked structure consisting of a hole-transporting layer/EML/electron-transporting layer is successfully fabricated. We also developed a series of sky-blue phosphorescent platinum(II) complexes bearing a hole-transporting dendron, and solution-processed multilayer OLEDs were successfully fabricated in a similar way to the dendritic iridium(III) emitters [39]. The bulkiness of the dendron-appended cyclometalated ligand and the ancillary ligand significantly affected excimer formation in the film state, and we obtained white electroluminescence (EL) from the fabricated device in which the monomer-based sky-blue emission and the excimer-based reddish orange emission are balanced to cover the whole visible region. Thus, phosphorescent organometallic complexes applicable to non-doped, solution-processed OLEDs should have great potential towards next-generation low-cost device fabrication in an industrial scene.

**Figure 1:**
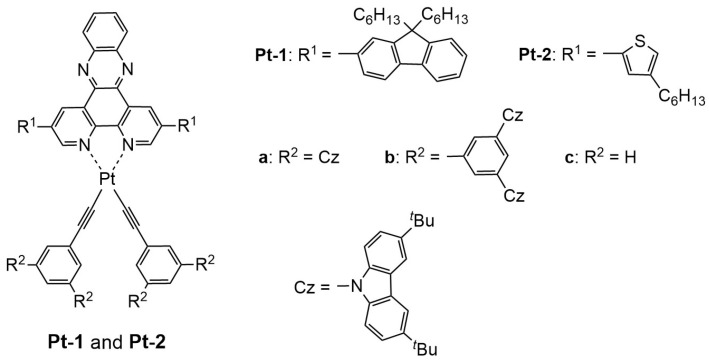
Chemical structures of phosphorescent platinum(II) complexes **Pt-1a**–**c** and **Pt-2a**–**c**.

In this study, we report a new type of phosphorescent platinum(II) complex for solution-processed OLEDs bearing a non-doped EML, namely, **Pt-1** (**Pt-1a** and **-1b**) and **Pt-2** (**Pt-2a**, and **-2b**), as shown in Figure 1. A dipyrido[3,2-*a*;2′3′-*c*]phenazine (dppz)–platinum(II)–acetylide complex is employed as a phosphorescent platform, which was previously reported by our group [40]. The electron-donating aromatic substituents at the 2,7-positions, such as fluorene and thiophene, are essential for room-temperature phosphorescence. On the phenylacetylide ligands, carbazole-based hole-transporting moieties are introduced to enhance charge carrier mobility in the EML, where peripheral lipophilic *tert*-butyls are attached to enhance film-forming ability [37,38,39]. Here, we report the synthesis and photoluminescence (PL) properties of these dppz–platinum(II)–phenylacetylide complexes and the fabrication of solution-processed OLEDs consisting of these complexes as a non-doped EML.

## 2. Results and Discussion

### 2.1. Synthesis

**Scheme 1:**
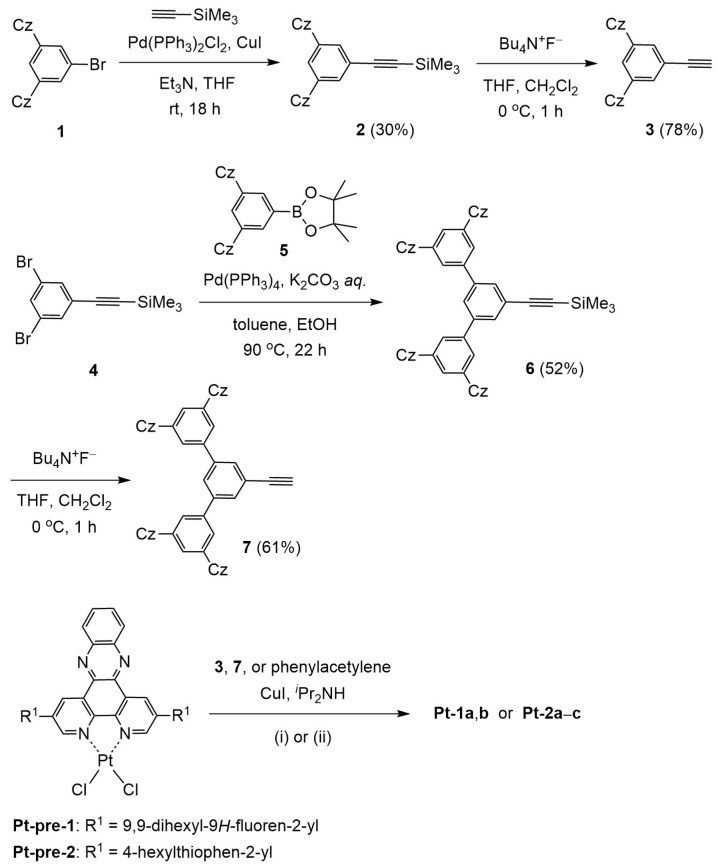
Syntheses of **Pt-1a**,**b** and **Pt-2a**–**c**. The substituent Cz represents the 3,6-di-*tert*-butyl-9*H*-carbazol-9-yl group. *Conditions*: (i) dichloromethane, rt (**Pt-1a**,**b** and **Pt-2 a**,**b**); (ii) toluene, 70 °C (**Pt-2c**).

Scheme 1 shows the synthetic schemes of **Pt-1a**,**b** and **Pt-2a**–**c**. The synthesis of **Pt-1c** has been reported previously [40]. The other complexes were prepared in a similar way to **Pt-1c** by the reaction of the corresponding dppz–PtCl_2_ precursors (**Pt-pre-1** [40] or **Pt-pre-2** (see Supplementary Materials)) with phenylacetylene derivatives (**3**, **7**, or phenylacetylene). First, the phenylacetylene derivatives bearing 3,6-di-*tert*-butylcarbazole-based hole-transporting moieties **3** and **7** were synthesized. The Sonogashira coupling reaction of **1** [39] with trimethylsilylacetylene followed by the desilylation with tetrabutylammonium fluoride afforded **3** [41] as the monodentate ligand for **Pt-1a** and **Pt-2a**. On the other hand, **7,** as the acetylide ligand for **Pt-1b** and **Pt-2b,** was prepared by the Suzuki–Miyaura cross-coupling reaction of **4** [42] with **5** [39], followed by the fluoride-promoted desilylation. We examined the ligand exchange reaction of precursor complexes **Pt-pre-1** and **Pt-pre-2** with the phenylacetylene derivatives to obtain **Pt-1** and **Pt-2**. The synthesis of **Pt-pre-1** has already been reported [40], and **Pt-pre-2** was prepared in a similar way from the corresponding dppz derivative and Pt(DMSO)_2_Cl_2_ (DMSO; dimethyl sulfoxide), as described in the Supplementary Materials. The reaction of **Pt-pre-1** or **Pt-pre-2** with **3** or **7** proceeded smoothly in 38–56% yields in the presence of reaction promotors copper(I) iodide and di-*iso*-propylamine. In this study, the reference complexes **Pt-1c** and **Pt-2c**, lacking 3,6-di-*tert*-carbazole-based hole-transporting moieties, were also employed. The complex **Pt-2c** was prepared by the reaction of **Pt-pre-2** with phenylacetylene in a 50% yield. The prepared dppz–platinum(II)–phenylacetylide complexes, except for **Pt-2c**, were characterized by ^1^H and ^13^C NMR and MALDI-TOF mass spectra as well as elemental analysis. In the case of **Pt-2c**, the very low solubility in any solvent prevented us from characterization by ^1^H and ^13^C NMR spectroscopy; thus, characterization of this compound was carried out by MALDI-TOF mass spectroscopy and elemental analysis.

### 2.2. UV-vis Absorption and Photoluminescence Properties

**Figure 2:**
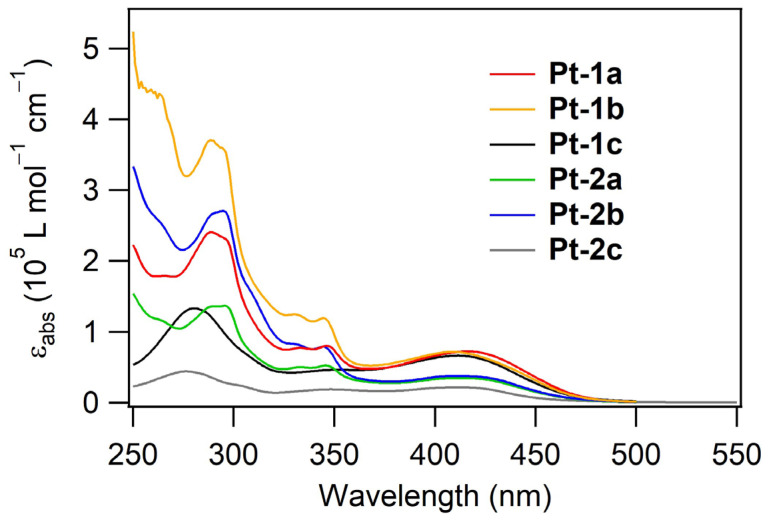
UV-vis absorption spectra of **Pt-1** and **Pt-2** in dichloromethane (10 μM) at rt.

Figure 2 shows the UV-vis absorption spectra of **Pt-1** and **Pt-2** in de-aerated dichloromethane (10 μM) at rt, and the spectral data are summarized in Table 1. The assignment of transition bands was carried out basically according to the previous report for **Pt-1c** [40]. The absorption bands ranging in the near UV region at less than ca. 350 nm are ascribed to π–π* and weak charge transfer transition bands on the dppz and carbazole-appended phenylacetylide ligands. A broad absorption band with a molar absorption coefficient (ε_abs_) of ca. 72,000 L mol^−1^ cm^−1^ was observed around 410 nm for each of **Pt-1a** and **Pt-1b**, which is assigned mainly to the intraligand charge transfer (ILCT) transition from the fluorenyl side-arms to the dppz core. For each of **Pt-2a** and **Pt-2b**, a similar absorption band with ε_abs_ of ca. 35,000–38,000 L mol^−1^ cm^−1^ was observed in the same region, assigned to the ILCT transition from the thienyl side-arms to the dppz core. Previously, we clarified that phosphorescence emission from **Pt-1c** is ascribed to the radiative process from the lowest triplet level (T_1_) to the ground-state singlet level (S_0_) consisting of metal-to-ligand charge-transfer (MLCT; dπ(Pt)-to-π*(dppz)) and ligand-to-ligand charge transfer (LLCT; π(phenylacetylide)-to-π*(dppz)) transitions [40]. Although the S_0_–T_1_ forbidden transition bands were not clearly observed, phosphorescent emission from the present platinum(II) complexes should be based on the MLCT and LLCT transitions. This assignment is supported by natural transition orbital (NTO) analyses based on time-dependent density-functional theory (TD-DFT) calculations for **Pt-1a**,**c** and **Pt-2a**,**c** (see Supplementary Materials).

**Figure 3:**
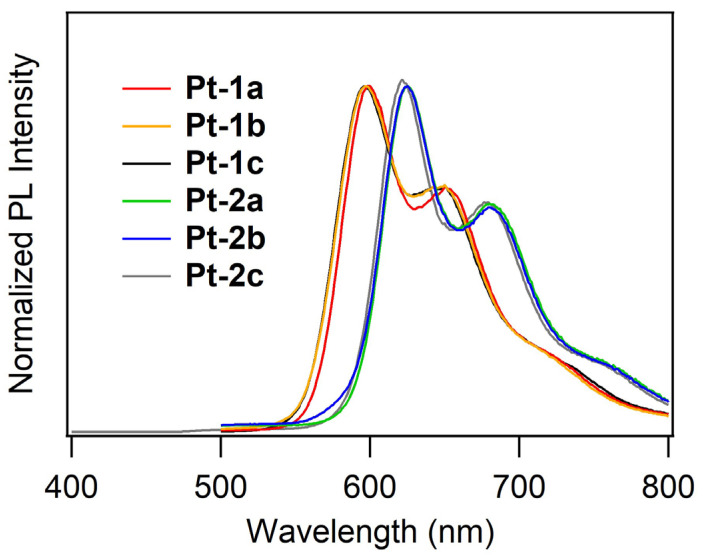
PL spectra of **Pt-1** and **Pt-2** in de-aerated dichloromethane (1.0 μM) at rt.

PL spectra of **Pt-1** and **Pt-2** in de-aerated dichloromethane (1 μM) at room temperature are shown in Figure 3. The PL lifetimes (τ_PL_) and PL quantum yields (*Φ*_PL_) of these complexes were also obtained, and these values allowed us to determine the radiative and non-radiative decay rate constants (*k*_r_ and *k*_nr_, respectively) of the photo-excited complexes. All the obtained data are summarized in Table 1. Contrary to the electronic absorption, the PL profile of **Pt-1** was quite different from that of **Pt-2**. The complexes **Pt-1a**–**c** exhibited reddish orange emission at 597–599 nm with *Φ*_PL_s of 0.033–0.18, the PL maxima (λ_PL_) of which are independent of the acetylide ligands. On the other hand, **Pt-2a**–**c** exhibited red emission at 622–625 nm with *Φ*_PL_s of 0.068–0.14, the λ_PL_s of which are ca. 25 nm red-shifted in comparison with those of **Pt-1a**–**c** under the same conditions. This red shift is obviously induced by introduction of the thienyl side-arms to the dppz-2,7 positions in place of the fluorenyl side-arms. The observed emission is based on phosphorescence because microsecond-order τ_PL_s (1.04–2.42 μs) were obtained, as seen in typical phosphorescent organoplatinum(II) complexes [11,15,43]. In the comparison of **Pt-1a** (or **Pt-2a**) and **Pt-1b** (or **Pt-2b**), the latter is more emissive, indicating that the *Φ*_PL_ is sensitive to the substituent(s) on the phenylacetylide ligand. Upon investigation of photokinetic parameters *k*_r_ and *k*_nr_, any tendencies were not found for the effect of the structure of the phenylacetylide ligand on the PL properties in solution at this point. However, **Pt-1b**, showing the highest *Φ*_PL_ among the present complexes (*Φ*_PL_: 0.18), afforded the largest *k*_r_ of 0.15 μs^−1^, whereas **Pt-1a**, showing the lowest *Φ*_PL_, provided the smallest *k*_r_ in spite of the relatively small *k*_nr_. The less emissive PL profile of **Pt-1a** is obviously responsible for the fact that the *k*_r_ of **Pt-1a** was one order of magnitude smaller than that of **Pt-1b**.

**Figure 4:**
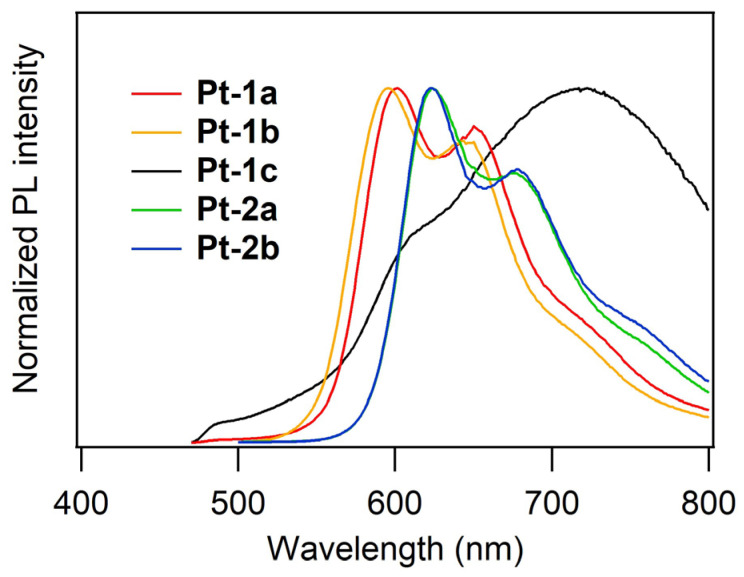
PL spectra of neat films of **Pt-1** and **Pt-2** at rt under nitrogen atmosphere. The spectrum for **Pt-2c** was not obtained because the low solubility in solvents prevented neat film formation.

The PL properties of the neat films of **Pt-1** and **Pt-2** were also evaluated under nitrogen atmosphere, as shown in Figure 4. The spectral data are summarized in Table 2. The sample films were prepared by a spin-coating method. Unfortunately, the sample film of **Pt-2c** was not obtained due to quite low solubility in any organic solvents. Although the reference complex **Pt-1c** exhibited reddish orange emission (λ_PL_: 597 nm) in solution, its neat film showed a broadened, red-shifted emission spectrum in the deep red-to-near infrared region (λ_PL_: 722 nm) with a very low PL intensity (*Φ*_PL_: 0.024). This indicates the aggregate or excimer formation of **Pt-1c** in the solid state. On the other hand, **Pt-1a** and **Pt-1b** exhibited monomer-based reddish orange emission at 595–596 nm in the film state, the spectra of which were consistent with those in solution. One can see that the steric hindrance of carbazole-appended phenylacetylide ligands prevents the aggregate formation of **Pt-1a**,**b** and **Pt-2a**,**b**. Regardless of the phenylacetylide ligands, **Pt-1a** and **Pt-1b** exhibited similar *Φ*_PL_s of 0.094 and 0.083, respectively. The neat films of **Pt-2a** and **Pt-2b** also exhibited red emission (λ_PL_: 622–624 nm) assigned to their monomeric forms, which is consistent with the emission in solution. Thus, the emission maxima of **Pt-2a** and **Pt-2b** in the film state were red-shifted by ca. 30 nm in comparison with those of **Pt-1a** and **Pt-1b**, as observed from their dichloromethane solutions. As for *Φ*_PL,_ in the film state, **Pt-2a** and **Pt-2b** are slightly less emissive (*Φ*_PL_: 0.042 and 0.069, respectively) than **Pt-1a** and **Pt-1b**. These results indicate that the present platinum(II) complexes bearing the carbazole-appended phenylacetylide ligands are expected to work as a non-doped EML for solution-processed OLEDs.

### 2.3. Evaluation of HOMO and LUMO Energy Levels

**Figure 5:**
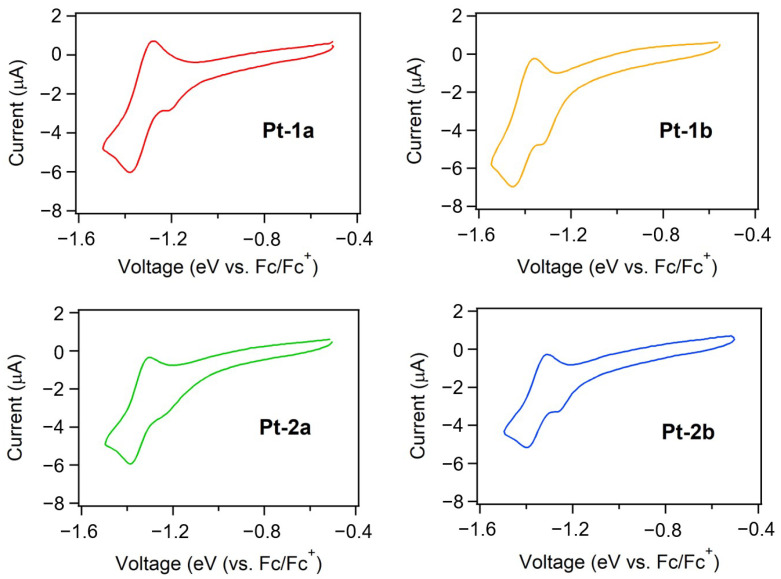
Cyclic voltammograms of **Pt-1a**,**b** and **Pt-2a**,**b** at rt. The electrochemical measurements were carried out in dichloromethane de-aerated by nitrogen gas bubbling, where 0.1 M tetrabutylammonium perchlorate was used as a supporting electrolyte. The reduction potentials were recorded relative to an Ag/AgNO_3_ reference electrode with a platinum wire used for both working and counter electrodes.

**Figure 6:**
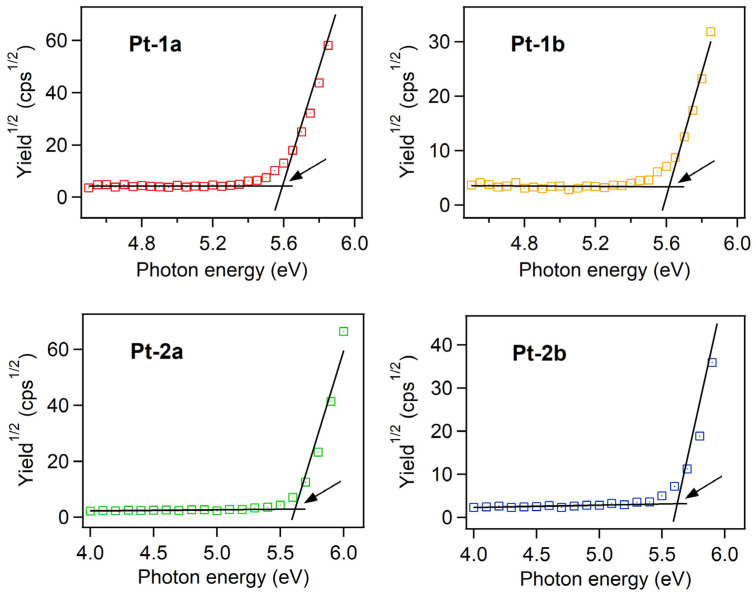
Photoelectron yield spectra of the neat films of **Pt-1a**,**b** and **Pt-2a**,**b** under ambient conditions. The arrows point to the onsets of the spectra corresponding to the ionization potentials.

The energy levels of the highest occupied and lowest unoccupied molecular orbitals (HOMO and LUMO, respectively) of an emitting material are closely related to charge carrier injection into the emitting center in an OLED. To determine HOMO and LUMO energy levels (*E*_HOMO_ and *E*_LUMO_, respectively) of **Pt-1a**,**b** and **Pt-2a**,**b**, their electrochemical properties were investigated. The obtained cyclic voltammograms are shown in Figure 5, and the data are summarized in Table 3. These complexes showed pseudo-reversible reduction cycles in which the half-wave reduction potentials vs. the ferrocene/ferrocenium (Fc/Fc^+^) redox couple, namely *E*_1/2,red_, were found at ca. −1.5 V, although an irreversible peak due to a small amount of residual oxygen was observed from −1.2 to −1.3 V. Thus, the *E*_LUMO_s were evaluated according to the Equation (1):

*E*_LUMO_ (eV) = − ((*E*_1/2,red_ − *E*_1/2,Fc_) + 4.80), (1)

where *E*_1/2,Fc_ is the redox potential for the Fc/Fc^+^ redox couple. As summarized in Table 3, the value of *E*_LUMO_ ranged from −3.29 to −3.31 eV. Unfortunately, any reversible oxidation cycles were not observed for **Pt-1a**,**b** and **Pt-2a**,**b**, and thus, *E*_HOMO_s were not obtained from the electrochemical measurement. Instead, these values were determined by the ionization potential (IP) measurement using ultraviolet photoelectron spectroscopy under ambient conditions. The photoelectron yield spectra of neat thin films of **Pt-1a**,**b** and **Pt-2a**,**b** are shown in Figure 6. As summarized in Table 3, *E*_HOMO_s were determined as ca. −5.6 eV from the onset energies of the spectra [44,45,46]. As reported previously, the HOMO and the LUMO are localized at the phenylacetylides and the dppz moiety, respectively [40], independent of the side-arms. Additionally, the electronic properties of the phenylacetylide ligands should be determined by the 3,5-bis(3,6-di-*tert*-butyl-carbazol-9-yl)phenyl substructures. Thus, both *E*_HOMO_ and *E*_LUMO_ are almost the same among **Pt-1a**,**b** and **Pt-2a**,**b**.

### 2.4. Fabrication of OLEDs

#### 2.4.1. Fabrication of Non-Doped OLEDs with a Simple Device Structure

**Figure 7:**
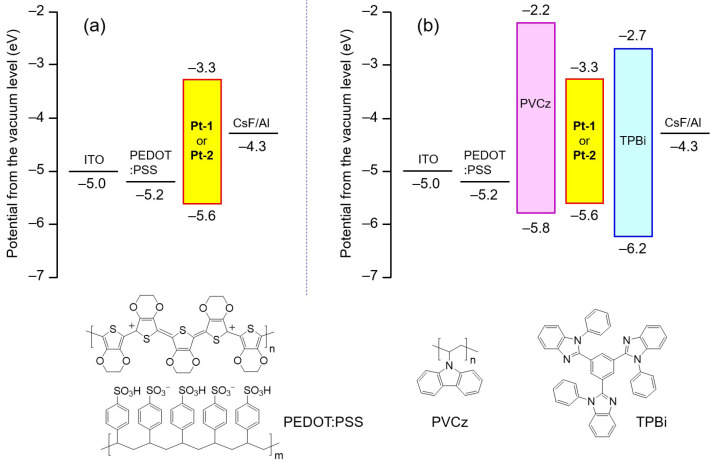
HOMO and LUMO levels of the constituent materials of (**a**) the devices S-1 and S-2 and (**b**) the devices M-1 and M-2. The chemical structures of PEDOT:PSS, PVCz, and TPBi are also illustrated therein.

Non-doped OLEDs employing a neat film of **Pt-1a**,**b** and **Pt-2a**,**b** as an EML were fabricated; namely, devices S-1a,b and S-2a,b, respectively. The device structure is as follows: ITO (transparent anode, 150 nm)/poly(3,4-ethylenedioxythiophene):poly(stylenesulfonate) (PEDOT: PSS, hole-injection layer, 40 nm)/EML (the platinum(II) complex, 50 nm)/cesium fluoride (CsF, electron-injection layer, 1.0 nm)/aluminum (Al, cathode, 250 nm). The PEDOT:PSS and EML were successively spin-coated onto ITO using a 2-propanol–water solvent mixture and toluene as ink solvents, respectively. The CsF and Al layers were placed on the EML by vacuum deposition. The HOMO and LUMO levels of the constituent materials for S-1 and S-2 are also shown in Figure 7a.

**Figure 8:**
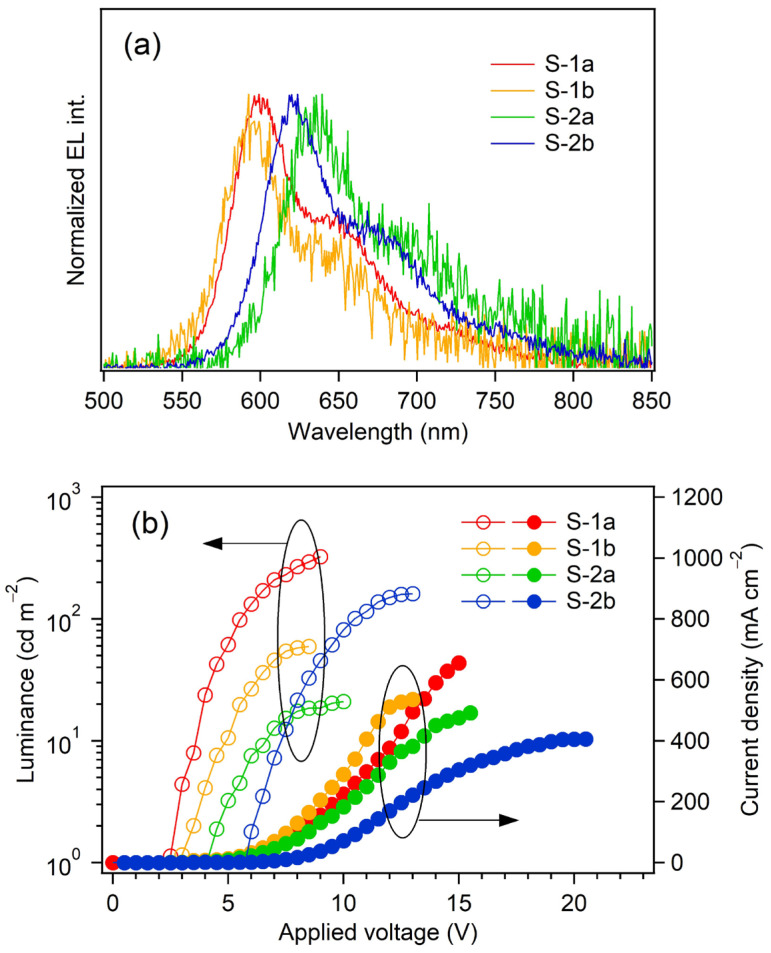
EL spectra at *L*_max_ (**a**) and *J*–*V*–*L* curves (**b**) of devices S-1 and S-2.

Figure 8 shows the EL spectra at the maximum luminance (*L*_max_) and the current density–voltage–luminance (*J*–*V*–*L*) relationships of S-1a,b and S-2a,b. The device performances are also summarized in Table 4. The devices S-1a and S-1b exhibited reddish orange EL upon application of a voltage higher than the turn-on voltage (*V*_on_: defined as the voltage where the luminance more than 1 cd m^−2^ is obtained), whereas S-2a and S-2b exhibited red EL. Relatively low *V*_on_s (2.5–5.5 V) were achieved because the direct injection of holes and electrons was possible on the single component EML from the anode–hole-injection layer and the cathode–electron-injection layer, respectively. The EL spectra were almost identical to the PL spectra of the constituent phosphorescent emitters: the emission maxima (λ_EL_) for S-1a and S-1b were observed at 599 and 592 nm, respectively, and those for S-2a and S-2b were observed at 639 and 624 nm, respectively. Obviously, the λ_EL_ of S-1a (S-2a) was slightly red-shifted in comparison with S-1b (S-2b): the electronic structure of the acetylide ligand should affect the EL spectrum slightly, although the reason is unclear at this point.

Among the fabricated devices, S-1a exhibited the highest *L*_max_ of 324 cd m^−2^ and the largest maximum external quantum efficiency (*η*_ext,max_) of 0.37%. The maximum current efficiency (*η*_j max_) and the maximum power efficiency (*η*_p max_) were also quite modest: 0.59 cd A^−1^ and 0.46 lm W^−1^, respectively. In this term, the values of the carrier balance (*γ*) were calculated for all the devices according to Equation (2):

*γ* (%) = 100 × [*η*_ext,max_/(*η*_ex_ × *Φ*_PL_ × *η*_ph_)], (2)

where *η*_ex_ and *η*_ph_ represent the light-extraction efficiency and the exciton formation efficiency, respectively. Supposing that *η*_ex_ and *η*_ph_ are 0.20 and 1.0, respectively, *γ* of S-1a was just 20%. The values of *γ* for the other devices were also very low (3.1–10%) in comparison with a theoretical maximum value of *γ* for a phosphorescent OLED (100%). Taking the device structure lacking an electron-transporting layer into consideration, such low device performance should be caused by the poor electron injection into the EML, in addition to the relatively low *Φ*_PL_s: holes are excessively injected in comparison with electrons (see Figure S1 in Supplementary Materials), and thus, a large part of the charge carrier recombination zone might be located outside the EML.

#### 2.4.2. Fabrication of Non-Doped OLED with a Multilayer Structure

Since the complexes **Pt-1a**,**b** and **Pt-2a**,**b** have four and eight lipo-soluble *tert*-butyl groups, respectively, they are soluble in cyclohexane and insoluble in protic solvents such as methanol. Thus, we were allowed to fabricate a multilayer-type, non-doped OLEDs bearing the platinum(II) complex-based EML by solution processing, aimed at the improvement of the device performance. The device structure is as follows: ITO (anode, 150 nm)/PEDOT: PSS (hole-injection layer, 40 nm)/poly(9-vinyl-9*H*-carbazole) (PVCz, 30 nm)/EML (30 nm)/1,3,5-tris(1-phenyl-1*H*-benzo[*d*]imidazol-2-yl)benzene (TPBi, 40 nm)/CsF (electron-injection layer, 1.0 nm)/Al (cathode, 250 nm). The fabricated multilayer devices are named M-1a,b and M-2a,b for **Pt-1a**,**b** and **Pt-2a**,**b**, respectively. The HOMO and LUMO levels of the constituent materials for M-1 and M-2 are also shown in Figure 7b. This multilayer device employed PVCz as a hole-transporting and electron-blocking layer and TPBi as an electron-transporting and hole-blocking layer [39]. The PVCz layer was spin-coated on the PEDOT:PSS layer using the toluene solution. Then, the emitting layer, i.e., **Pt-1** (or **Pt-2**), was successfully spin-coated onto the PVCz layer using the cyclohexane solution, because PVCz is insoluble in cyclohexane. Thereafter, the TPBi layer was spin-coated on the EML using the methanol solution. As the platinum(II) complexes were insoluble in methanol, it was successfully prepared without rinsing away the EML beneath. Indeed, we carried out a solvent-resistance test by rinsing the thin films of the present platinum(II) complexes with methanol. The thickness of each film was the same even after rinsing with methanol. Thus, from the utilization of the orthogonal solvent system, the multi-stacked organic layers were obtained. Finally, the CsF layer and the Al cathode were placed by vacuum deposition.

As shown in Figure 7b, the holes injected from the ITO anode (work function: −5.0 eV [28]) move to the EML of **Pt-1** or **Pt-2** (*E*_HOMO_: ca. −5.6 eV) through the PEDOT:PSS (*E*_HOMO_: −5.2 eV [28]) and PVCz (*E*_HOMO_: −5.8 eV [37]) layers, and then are blocked at the interface between the EML and the TPBi layer (*E*_HOMO_: −6.2 eV [28]) due to the relatively large *E*_HOMO_ gap (0.6 eV) between the platinum(II) complexes and TPBi. The electrons injected from the cathode move to the EML (*E*_LUMO_: −3.3 eV), through the LUMO level of TPBi (*E*_LUMO_: −2.7 eV [28]), and are blocked at the interface between the EML and the PVCz layer because the *E*_LUMO_ of PVCz (−2.2 eV [37]) is ca. 1.1 eV higher than that of **Pt-1** (or **Pt-2**). Therefore, the injected holes and electrons should be encapsulated effectively in the EML, and therein efficient hole–electron charge recombination should occur to generate efficiently the excitons of the phosphorescent platinum(II) complexes.

**Figure 9:**
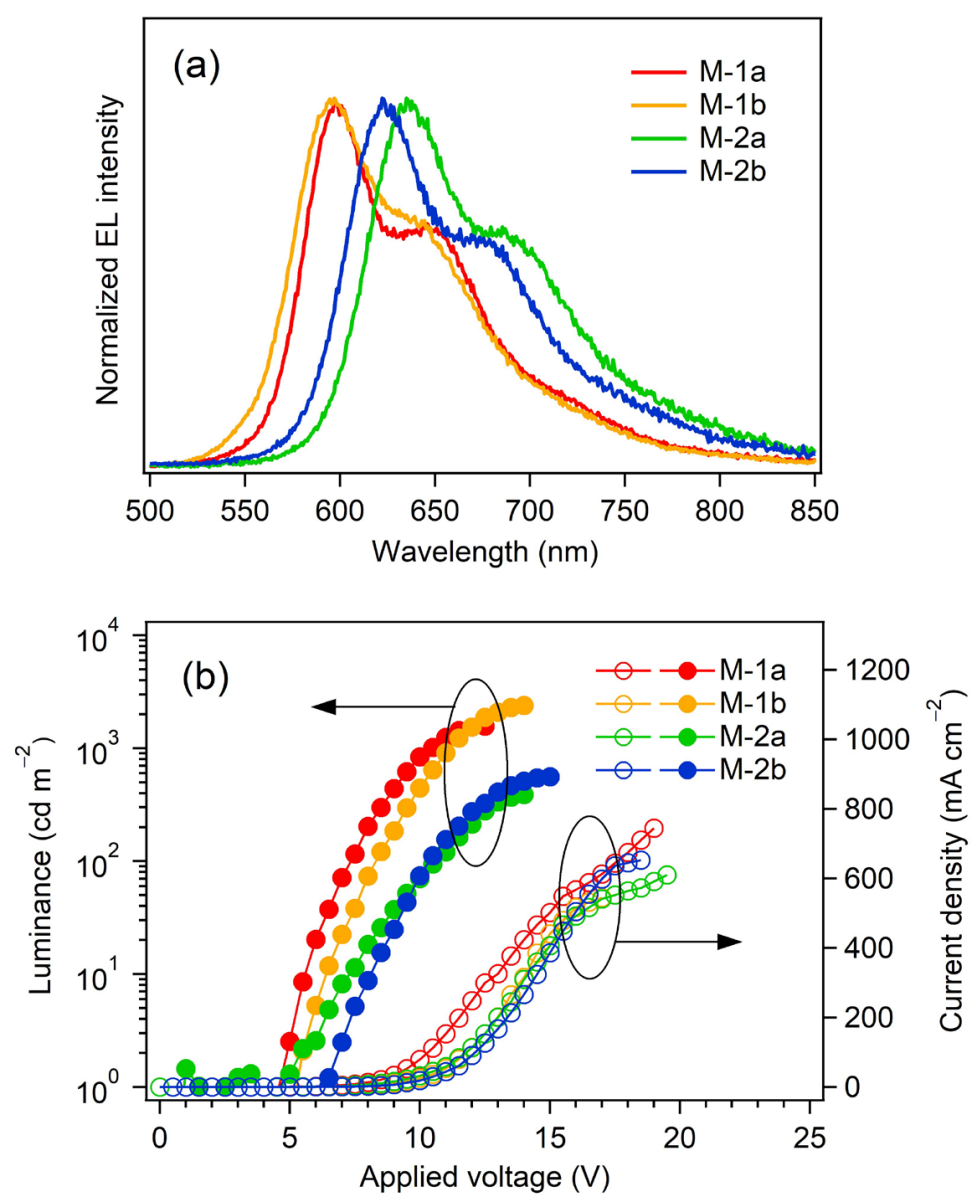
EL spectra at *L*_max_ (**a**) and *J*–*V*–*L* curves (**b**) of devices M-1 and M-2.

Figure 9 shows the EL spectra and current density–voltage–luminance (*J*–*V*–*L*) relationships of the devices M-1a,b and M-2a,b. The device performances are also summarized in Table 5. Reddish orange EL around 600 nm and red EL around 625 nm were observed for the devices M-1a,b and M-2a,b, respectively, and the EL spectra were identical to the EL spectra of the single-layer-type OLEDs consisting of **Pt-1** and **Pt-2**. The *V*_on_s of the present multilayer devices are slightly larger than those of the single-layer-type devices. One might see that the relatively large *V*_on_s are caused by the additional hole- and electron-transporting layers that enlarge the potentials of charge carrier injection into the EML in comparison with S-1 and S-2, as shown in Figure 7b. Obviously, the device performances were drastically improved: *L*_max_ of 391–1566 cd m^−2^, *η*_j max_ of 0.28–1.54 cd A^−1^, and *η*_p max_ of 0.012–0.67 lm W^−1^ were achieved. In particular, the device performances of M-1a and M-1b were better than those of M-2a and M-2b, and *η*_ext, max_s of ca. 1% were achieved by using **Pt-1a** and **Pt-1b** as the EML. Thus, the multilayer-type non-doped OLEDs are effective in achieving high device performance. This should be because of the improvement of the carrier balance in comparison with the single-layer-type OLED for each complex. Indeed, higher *γ* values (Table 5, 27.7–50.2%) were obtained in comparison with S-1a and S-1b, indicating that holes and electrons are confined in the EML more efficiently than the single-layer-type devices (see Figure S2 in Supplementary Materials).

Although the multilayer device structure allowed us to achieve the charge carrier injection into the EML and their confinement within the EML to improve the charge carrier balance, M-1a,b and M-2a,b still showed poorer device performance in comparison with reported phosphorescent OLEDs [36,37,39,47,48,49], whose EMLs were fabricated by solution-processing (Table 6). Obviously, the present devices are inferior to the examples in Table 6. This is due to the low *Φ*_PL_ as well as the low γ. These two factors should be improved towards practical uses.

## 3. Methods

### 3.1. Evaluation of Optical and Photophysical Properties

UV-vis absorption spectra were recorded on a Shimadzu (Tokyo, Japan) UV-3600 spectrophotometer using a quartz cell for solutions or a quartz plate for thin films. PL spectra were recorded on a Horiba Jobin Yvon Fluorog-3 spectrophotometer (Palaiseau. France). PL lifetimes were obtained on a Horiba Jobin Yvon FluoroCube spectroanalyzer, using a 390 nm nanosecond-order LED light source. PL quantum yields were measured on a Hamamatsu Photonics (Fukuoka, Japan) C9920 absolute PL quantum yield measurement system. The spectroscopic measurements were carried out immediately after the preparation of the samples. To avoid the influence of oxygen on the triplet excited state of the platinum(II) complexes, the sample solutions were deaerated by gentle nitrogen gas bubbling in a quartz cuvette for about 5 min, followed by complete sealing. Neat films of the platinum(II) complexes were prepared by spin coating from toluene solutions onto a quartz plate, and then dried at 120 °C for 1 h under an argon atmosphere. To avoid the influence of oxygen, the spectroscopic measurements of the neat films were carried out in a sample compartment filled with nitrogen gas.

### 3.2. Evaluation of Electrochemical Properties

Cyclic voltammograms were recorded on a Hokuto Denko (Tokyo, Japan) HZ-5000 electrochemical measurement system at a scanning rate of 200 mV/s. Electrochemical measurements were carried out in anhydrous dichloromethane, where 0.1 M tetrabutylammonium perchlorate was used as a supporting electrolyte. The reduction potentials were recorded relative to a Ag/AgNO_3_ reference electrode with a platinum wire used for both working and counter electrodes. All electrochemical experiments were performed at rt after degassing with argon gas for 2 min.

### 3.3. IP Measurements

The ionization potentials (IPs) of **Pt-1a**,**b** and **Pt-2a**,**b** were estimated by ultraviolet photoelectron spectroscopy under ambient conditions, using a Riken Keiki (Sakurai-shi, Japan) model AC-2 photoelectron spectrometer. The samples for the IP measurements were prepared on ITO–glass substrates by spin-coating from toluene solutions of the platinum(II) complexes.

### 3.4. Fabrication of OLEDs

#### 3.4.1. Fabrication of a Single-Layer Device

The pre-patterned ITO–glass substrate as an anode was routinely cleaned by ultrasonic treatment in detergent solution, water, acetone, chloroform, hexane, and 2-propanol, followed by drying with a gentle flow of argon gas. Then, the cleaned ITO substrate was subjected to UV–O_3_ treatment. Onto the ITO surface, a 2-propanol-water suspension of PEDOT:PSS (Clevios P CH 8000, 40 nm) was spin-coated as a hole-injection layer, and the prepared film was dried at 115 °C for 1 h. For fabrication of an EML (50 nm), **Pt-1** (or **Pt-2**) in toluene was filtered through a 0.2 μm Millex-FG filter (Millipore Co., Burlington, MA, USA), and the filtrate was spin-coated, followed by drying at 120 °C for 1 h under argon atmosphere. CsF (1.0 nm, purchased from Alfa Aesar, Ward Hill, MA, USA) and Al (250 nm, purchased from The Niraco Corporation, Washington, DC, USA) layers were successively vacuum-deposited onto the organic layer as an electron-injection layer and a cathode, respectively. Finally, the device was covered with a glass cap and encapsulated by a UV-curing epoxy resin under an argon atmosphere to keep the device away from air and moisture. The emitting area was 10 mm^2^. Device fabrication was carried out in a glove box filled with dry argon gas.

#### 3.4.2. Fabrication of a Multilayer Device

The pre-patterned ITO–glass substrate was treated for cleansing according to the same procedure on the fabrication of the above-mentioned single-layer device. The PEDOT:PSS layer (40 nm) was also routinely prepared by a spin-coating method. The PVCz layer (30 nm, purchased from Sigma-Aldrich, *M*_n_ 25,000–50,000, purified by reprecipitation from THF–methanol) was spin-coated from a toluene solution onto the PEDOT:PSS layer as a hole-transporting and electron-blocking layer, and then dried at 120 °C for 1 h. Then, **Pt-1** (or **Pt-2**) in cyclohexane was filtered through a 0.2 μm Millex-FG filter (Millipore Co.) and then the filtrate was spin-coated onto the PVCz layer as an EML (30 nm), followed by drying at 100 °C for 1 h under an argon atmosphere. Subsequently, TPBi (40 nm, purchased from Luminescence Technology, Palo Alto, CA, USA) was spin-coated from a methanol solution as an electron-transporting and hole-blocking layer and then dried at 80 °C for 10 min. CsF (1.0 nm) and Al (80 nm) layers were successively vacuum-deposited onto the TPBi layer. Finally, the device was covered with a glass cap and encapsulated by a UV-curing epoxy resin under an argon atmosphere to keep the device away from air and moisture. The area of the emitting part was 10 mm^2^. The device fabrication was carried out in a glove box filled with dry argon gas.

## 4. Conclusions

We synthesized novel phosphorescent dppz–platinum(II)–phenylacetylide complexes **Pt-1a**,**b** and **Pt-2a**,**b**, which possess hole-transporting dendrons on their phenylacetylide ligands. Appending fluoren-2-yl side-arms to the 2,7-positions of dppz, **Pt-1** series showed reddish orange phosphorescent emission in dichloromethane (λ_PL_: ca. 600 nm, *Φ*_PL_: 0.033–0.18). In the thin film state, **Pt-1a**,**b** showed PL spectra comparable to those in solution (λ_PL_: ca. 600 nm, *Φ*_PL_: ca. 0.09), although the neat film of the reference complex with unsubstituted phenylacetylides **Pt-1c** exhibited weak near-infrared PL (λ_PL_: 722 nm, *Φ*_PL_: 0.024) due to aggregate or excimer emission. Additionally, the thiophene side-arm-appended **Pt-2** series exhibited red phosphorescent emission in the film state (λ_PL_: ca. 625 nm, *Φ*_PL_: 0.042–0.069), similar to their PL spectral profile in solution. Thus, the introduction of the 3,6-di-*tert*-butylcarbaole-functionalized phenylacetylides to the platinum center effectively suppressed intermolecular interactions at the ground and/or excited state, facilitating the non-radiative decay process. Employing thin films of **Pt-1a**,**b** and **Pt-2a**,**b** as an EML, non-doped OLEDs were successfully fabricated by solution-processing, which gave EL based on PL of the neat film of the corresponding platinum(II) complex. The devices with only the EML between hole- and electron-injecting layers showed deteriorated device performance due to a low charge carrier balance (*γ*: 3.1–20%). On the other hand, by employing the multilayer devices with hole- and electron-transporting layers on the anodic and cathodic interfaces of the EML, respectively, the value of *γ* was improved to 28–50% for each of **Pt-1a**,**b** and **Pt-2a**,**b**. Such devices consisting of layer-by-layer organic thin films were successfully fabricated by using an orthogonal solvent system. Therefore, the present study proposes a rational molecular design of charge carrier-transporting phosphorescent platinum(II) complexes for non-doped OLEDs fabricated by solution-processing. Towards application in practical situations, device performance should be further improved. More sophisticated ligand designs of dppz and charge carrier-transporting acetylides should bring about an increase in *Φ*_PL_, and the carrier balance *γ* is expected to be improved by employing a better-designed device configuration. Further investigation to optimize the molecular design and the device configuration is ongoing.

## Figures and Tables

**Table 1:** UV-vis absorption and PL spectral data of **Pt-1** and **Pt-2** in dichloromethane at room temperature.

Compd	λ_abs_/nm (log [ε_abs_/(L mol^−1^ cm^−1^)]) ^1^	λ_PL_/nm ^1,2^	*Φ*_PL_ ^1,2^	τ_PL_/μs ^3^	*k*_r_/μs^−1^ ^4^	*k*_nr_/μs^−1^ ^5^
**Pt-1a**	289 (5.38), 346 (4.91), 416 (4.86)	599, 653	0.033	2.36	0.014	0.41
**Pt-1b**	289 (5.56), 344 (5.08), 408 (4.86)	597, 650	0.18	1.18	0.15	0.69
**Pt-1c**	280 (5.12), 411 (4.82)	597, 650	0.14	2.31	0.061	0.37
**Pt-2a**	295 (5.14), 346 (4.72), 413 (4.54)	625, 680	0.068	1.04	0.065	0.90
**Pt-2b**	295 (5.43), 344 (4.90), 410 (4.58)	625, 680	0.11	2.42	0.045	0.37
**Pt-2c**	276 (4.43), 412 (2.15)	622, 679	0.14	1.63	0.083	0.53

^1^ The concentration was adjusted to 10 μM for UV-vis and 1 μM for PL. For PL, the samples were de-aerated by gentle nitrogen gas bubbling followed by complete sealing. ^2^ Excited at the longest λ_PL_. The values of *χ*^2^ ranged from 1.00 to 1.06. ^3^ Excited at 390 nm and detected at the shorter λ_PL_. ^4^ *k*_r_ = *Φ*_PL_/τ_PL_. ^5^ *k*_nr_ = (1 − *Φ*_PL_)/τ_PL_.

**Table 2:** PL properties of neat thin films of **Pt-1** and **Pt-2** at rt.

Compd	λ_PL_/nm	*Φ*_PL_
**Pt-1a**	601, 650	0.094
**Pt-1b**	596, 643	0.083
**Pt-1c**	722	0.024
**Pt-2a**	624, 676	0.042
**Pt-2b**	622, 677	0.069
**Pt-2c**	N.D. ^1^	N.D. ^1^

^1^ Not determined due to low solubility in the solvent for spin-coating.

**Table 3:** Energy levels of HOMO (*E*_HOMO_) and LUMO (*E*_LUMO_) and HOMO–LUMO energy gaps (*E*_gap_) of **Pt-1a**,**b** and **Pt-2a**,**b**.

Compd	*E*_HOMO_ ^1^	*E*_LUMO_ ^2^	*E*_gap_ ^3^
**Pt-1a**	−5.59	−3.31	2.28
**Pt-1b**	−5.61	−3.30	2.31
**Pt-2a**	−5.61	−3.30	2.31
**Pt-2b**	−5.62	−3.29	2.33

^1^ The values of *E*_HOMO_ were determined by the IP measurements. ^2^ *E*_LUMO_ = −((*E*_1/2,red_ − *E*_1/2,Fc_) + 4.80). The values of *E*_1/2,red_ and *E*_1/2,Fc_ were obtained by the cyclic voltammetry measurements. ^3^ *E*_gap_ = *E*_LUMO_ − *E*_HOMO_.

**Table 4:** Device characteristics of devices S-1a,b and S-2a,b.

OLED	EML	*V*_on_ ^1^ /V	λ_EL_ /nm	*L*_max_ ^2^ /cd m^−2^	*η*_j max_ ^3^ /cd A^−1^	*η*_p max_ ^4^ /lm W^−1^	*η*_ext max_ ^5^ /%	CIE (x, y) ^6^	*γ* ^7^ /%
S-1a	**Pt-1a**	2.5	599	324	0.59	0.46	0.37	(0.61, 0.39)	20
S-1b	**Pt-1b**	3.0	592	59.4	0.096	0.059	0.052	(0.59, 0.42)	3.1
S-2a	**Pt-2a**	4.0	639	21.0	0.039	0.027	0.062	(0.67, 0.32)	7.4
S-2b	**Pt-2b**	5.5	624	162	0.13	0.053	0.14	(0.66, 0.34)	10

^1^ Turn-on voltage at 1 cd m^−2^. ^2^ Maximum luminance. ^3^ Maximum current efficiency. ^4^ Maximum power efficiency. ^5^ Maximum external quantum efficiency. ^6^ Commission internationale de l’éclairage (CIE) chromaticity coordinate. ^7^ Carrier balance for EL.

**Table 5:** Device characteristics of devices M-1a,b and M-2a,b.

OLED	EML	*V*_on_ ^1^ /V	λ_EL_ /nm	*L*_max_ ^2^ /cd m^−2^	*η*_j max_ ^3^ /cd A^−1^	*η*_p max_ ^4^ /lm W^−1^	*η*_ext max_ ^5^ /%	CIE (x, y) ^6^	*γ* ^7^ /%
M-1a	**Pt-1a**	4.5	597	1570	1.54	0.67	1.02	(0.60, 0.39)	43
M-1b	**Pt-1b**	5.0	597	2390	1.77	0.60	1.04	(0.58, 0.40)	50
M-2a	**Pt-2a**	5.0	635	391	0.28	0.13	0.44	(0.65, 0.31)	42
M-2b	**Pt-2b**	6.0	622	561	0.39	0.12	0.48	(0.62, 0.32)	28

^1^ Turn-on voltage at 1 cd m^−2^. ^2^ Maximum luminance. ^3^ Maximum current efficiency. ^4^ Maximum power efficiency. ^5^ Maximum external quantum efficiency. ^6^ Commission internationale de l’éclairage (CIE) chromaticity coordinate. ^7^ Carrier balance for EL.

**Table 6:** Examples of phosphorescent OLEDs whose emitting layers were fabricated by solution-processing.

Device Structure	EML	Processing for EML	*η*_ext_ ^1^ /%	Reference
ITO/PEDOT:PSS/EML/B3PYMPM ^2^/LiF/Al	Dendritic iridium(III) emitter, Green EL	Solution, Non-doped	8.3 ^13^	36
ITO/PEDOT:PSS/PVCz/EML/BPOPB/LiF/Al	Dendritic iridium(III) emitter, Sky-blue EL	Solution, Non-doped	5.72 ^14^	37
ITO/PEDOT:PSS/PVCz/EML/TPBi/LiF/Al	Platinum(II) emitter bearing hole-transporting units, Reddish Orange Excimer EL	Solution, Non-doped	6.5 ^14^	39
ITO/PEDOT:PSS/EML/TPBi/LiF/Al	Ir(piq)_3_ ^7^ doped into NPB ^8^:TPBi, Red EL	Solution, Doped	15.1 ^15^	47
ITO/PEDOT:PSS/TCTA ^3^/EML/TPBi/LiF/Al	Ir(piq)_2_acac ^9^ doped into 2-TNATA ^10^:TPBi, Red EL	Solution, Doped	7.7	48
ITO/PEDOT:PSS/EML/DPEPO ^4^/TmPyPB ^5^/Liq ^6^/Al	mCP ^11^:Ir(III) phosphor ^12^, Red EL	Solution, Doped	5.94 ^14^	49
ITO/PEDOT:PSS/EML/TPBi/CsF/Al (Device M-1b)	**Pt-1a**	Solution, Non-doped	1.04	This work

^1^ External quantum efficiency. ^2^ Bis-4,6-(3,5-di-3-pyridylphenyl)-2-methylpyrimidine. ^3^ 4,4,4-Tris(*N*-carbazolyl)triphenylamine. ^4^ (Oxybis(2,1-phenylene))bis(diphenylphosphine oxide). ^5^ 3,3′-(5′-(3-(Pyridin-3-yl)phenyl)-[1,1′:3′,1″-terphenyl]-3,3″-diyl)dipyridine. ^6^ (8-Quinolinolato)lithium. ^7^ Tris(2-phenylisoquinolinato-*N*,*C*^2′^)Ir(III). ^8^ *N*,*N*’-bis(1-naphtalen-1-yl)-*N*,*N*’-diphenylbenzidine. ^9^ Bis(2-phenylisoquinolinato-*N*,*C*^2′^)Ir(III) acetylacetonate. ^10^ 4,4′,4″-Tris(*N*-(2-naphthyl)-*N*-phenylamino)triphenylamine. ^11^ 1,3-Bis(carbazol-9-yl)benzene. ^12^ Bis [2-(9,10-dioxoanthracen-2-yl)isoquinolinato-*N*,*C*^3′^]iridium(III) acetylacetonate. ^13^ Obtained at 100 cd m^−2^. ^14^ Maximum value. ^15^ Value at the highest luminous efficiency.

## Data Availability

The original data presented in this study are included in the main text and the Supplementary Materials. Further inquiries can be directed to the corresponding author.

## Supplementary Materials

The following supporting information can be downloaded at: https://www.mdpi.com/article/10.3390/molecules29163849/s1, Materials Preparation; Figure S1: Schematic illustration for the device working mechanism of the present single-layer-type OLED; Figure S2: Schematic illustration for the device working mechanism of the present multilayer OLED; Figure S3: ^1^H and ^13^C NMR spectra of **2**; Figure S4: ^1^H and ^13^C NMR spectra of **3**; Figure S5: ^1^H and ^13^C NMR spectra of **6**; Figure S6: ^1^H and ^13^C NMR spectra of **7**; Figure S7: ^1^H and ^13^C NMR spectra of 2,7-bis(4-hexylthiophen-2-yl)dipyrido[3,2-*a*:2′,3′-*c*]phenazine; Figure S8: ^1^H NMR spectrum of **Pt-pre-2**; Figure S9: ^1^H and ^13^C NMR spectra of **Pt-1a**; Figure S10: ^1^H and ^13^C NMR spectra of **Pt-1b**; Figure S11: ^1^H and ^13^C NMR spectra of **Pt-2a**; Figure S12: ^1^H and ^13^C NMR spectra of **Pt-2b**; Figure S13; Natural transition orbitals (NTO) analyses for **Pt-1a**,**c** and **Pt-2a**,**c**.
