# Supplementary material for: Synthesis, Photoluminescence, and Electroluminescence of Phosphorescent Dipyrido[3,2-a;2′3′-c]phenazine–Platinum(II) Complexes Bearing Hole-Transporting Acetylide Ligands
Source: Molecules. 2024 Aug 14;29(16):3849. doi: 10.3390/molecules29163849 (PMC11356835; doi:10.3390/molecules29163849)
Supplement: Supplementary file 1 [file molecules-29-03849-s001.zip › molecules-3106491-supplementary.pdf]

Supplementary Materials

Synthesis, Photoluminescence, and Electroluminescence of Phosphorescent Dipyrido[3,2-*a*;2′3′-*c*]phenazine–Platinum(II) Complexes Bearing Hole-Transporting Acetylide Ligands

Hiroki Matsuura [1], Naoki Okamura [1], Masaki Nagaoka [2], Naoya Suzuki [1,2], Shintaro Kodama [1,2], Takeshi Maeda [1,2] and Shigeyuki Yagi [1,2,*]

[1] Department of Applied Chemistry, Graduate School of Engineering, Osaka Prefecture University, 1-1 Gakuen-cho, Naka-ku, Sakai 599-8531, Osaka, Japan; naoki_okamura@osakafu-u.net (N.O.); nsuzuki@omu.ac.jp (N.S.); skodama@omu.ac.jp (S.K.); tmaeda@omu.ac.jp (T.M.)

[2] Department of Applied Chemistry, Graduate School of Engineering, Osaka Metropolitan University, 1-1 Gakuen-cho, Naka-ku, Sakai 599-8531, Osaka, Japan; su23437o@st.omu.ac.jp

* Correspondence: yagi@omu.ac.jp

Table of Contents

1. Materials Preparation

1.1. General Procedures

1.2. Preparation of Materials

2. Device Working Mechanisms

2.1. Single-Layer-Type Device

Figure S1: Schematic illustration for device working mechanism of the present single-layer-type OLED.

2.2. Multilayer Device

Figure S2: Schematic illustration for device working mechanism of the present multilayer OLED.

3. ^{1}H and ^{13}C NMR Spectra

Figure S3: ^{1}H and ^{13}C NMR spectra of **2.**

Figure S4: ^{1}H and ^{13}C NMR spectra of **3.**

Figure S5: ^{1}H and ^{13}C NMR spectra of **6.**

Figure S6: ^{1}H and ^{13}C NMR spectra of **7.**

Figure S7: ^{1}H and ^{13}C NMR spectra of 2,7-bis(4-hexylthiophen-2-yl)dipyrido[3,2-*a*:2',3'-*c*]-

phenazine.

Figure S8: ^{1}H NMR spectrum of **Pt-pre-2.**

Figure S9: ^{1}H and ^{13}C NMR spectra of **Pt-1a.**

Figure S10: ^{1}H and ^{13}C NMR spectra of **Pt-1b.**

Figure S11: ^{1}H and ^{13}C NMR spectra of **Pt-2a.**

Figure S12: ^{1}H and ^{13}C NMR spectra of **Pt-2b.**

4. Natural Transition Orbital (NTO) Analyses

Figure S13: Natural transition orbital (NTO) analyses for **Pt-1a**,**c** and **Pt-2a**,**c.**

1. Materials Preparation

1.1. General Procedures

^{1}H NMR (400 MHz) and ^{13}C NMR (100 MHz) spectra were obtained on a JEOL JNM-ECX400 or a JEOL JNM-ECS400 spectrometer, using tetramethylsilane (0.00 ppm) and residual $CDHCl_2$ (5.32 ppm) as an internal standard for chloroform-*d* and dichloromethane-d_2, respectively. ^{13}C NMR spectra were obtained on the same spectrometer as ^{1}H NMR, using residual $CHCl_3$ (77.16 ppm) as an internal standard for chloroform-*d*. Matrix-assisted laser desorption/ionization time-of-flight (MALDI-TOF) mass spectra were measured on a Shimadzu-Kratos AXIMA-CFR PLUS TOF mass spectrometer, using α-cyano-4-hydroxycinnamic acid (CHCA) as a matrix. Elemental analyses were carried out on a J-Science MICRO CORDER JM10 analyzer. The compounds **1** [S1], **4** [S2], and **5** [S1] were prepared according to the literature. Although the synthetic procedures for **2** and **3** were previously reported [S3], they were prepared in this study according to modified procedures. As catalysts for the cross-coupling reactions, $Pd(PPh_3)_4$ and $Pd(PPh_3)_2Cl_2$ were purchased from WAKO Pure Chemical Industries, Ltd. The reagents and solvents required for the material preparations were purchased from WAKO Pure Chemical Industries, Ltd.; Tokyo Chemical Industry Co., Ltd.; and Sigma-Aldrich Co. and used as purchased. Dry solvents were also purchased when necessary.

1.2. Preparation of Materials

*9,9'-(5-((Trimethylsilyl)ethynyl)-1,3-phenylene)bis(3,6-di-tert-butyl-9H-carbazole) (**2**).*
A mixture of **1** (3.56 g, 5.00 mmol), $Pd(PPh_3)_2Cl_2$ (70.1 mg, 0.100 mmol), and CuI (37.8 mg, 0.198 mmol) in asolvent mixture of dry THF (10 mL) and triethylamine (1.1 mL) was stirred at rt under a nitrogen atmosphere. Then, trimethylsilylacetylene (0.88 mL, 0.61 g, 6.2 mmol) was added to the reaction mixture slowly. The reaction mixture gradually turned dark. The reaction mixture was stirred for 18 h at rt. The solvent was removed by evaporation, and the residue was purified by silica column chromatography, eluting with chloroform–hexane (1:1, v/v) to yield a crude solid product. Recrystallization from chloroform–methanol afforded **2** as a white powder (1.08 g, 1.48 mmol, 30%). Characterization data of ^{1}H and ^{13}C NMR were identical to those in the literature [S3]; ^{1}H NMR (400 MHz, $CDCl_3$) δ 0.27 (s, 9H), 1.46 (s, 36H), 7.45 (d, *J* = 8.8 Hz, 4H), 7.49 (dd, *J* = 1.8 and 8.8 Hz, 4H), 7.73 (d, *J* = 1.8 Hz, 2H), 7.75 (t, *J* = 1.8 Hz, 1H), 8.13 (d, *J* = 1.8 Hz, 4H); ^{13}C NMR (100 MHz, $CDCl_3$) δ 0.15, 32.14, 34.93, 97.01, 103.35, 109.30, 116.54, 123.82, 124.00, 124.87, 126.47, 128.25, 138.94, 139.92, 143.58; MALDI-TOF MS (*m*/*z*): M^+ calcd for $C_{51}H_{60}N_2Si$, 728.45; found, 728.46. Anal. Calcd for $C_{51}H_{60}N_2Si$: C, 84.01; H, 8.29; N, 3.84. Found: C, 83.77; H, 8.06; N, 3.77.

*9,9'-(5-Ethynyl-1,3-phenylene)bis(3,6-di-tert-butyl-9H-carbazole) (**3**).*
A mixture of **2** (624 mg, 0.856 mmol) and tetrabutylammonium fluoride (2.24 g, 8.57 mmol) in a solvent micture of THF (8.5 mL) and dichloromethane (85 mL) was stirred for 1 h. The solvent was removed on a rotary evaporator. The residue was dissolved in chloroform (200 mL) and then the solution was washed with water (100 mL × 2) and sat. brine (100 mL). The obtained organic solution was dried over anhydrous $MgSO_4$. The solvent was removed on a rotary evaporator and the residue

was purified by silica gel column chromatography using chloroform–hexane (1:4, v/v) as an eluent to obtain **3** as a white powder (0.440 g, 4.60 mmol, 78%). Characterization data of ^{1}H and ^{13}C NMR were identical to those in the literature [S3]; ^{1}H NMR (400 MHz, $CDCl_3$) δ 1.46 (s, 36H), 3.23 (s, 1H), 7.45 (d, *J* = 8.8 Hz, 4H), 7.49 (dd, *J* = 1.8 and 8.8 Hz, 4H), 7.76 (d, *J* = 2.2 Hz, 2H), 7.81 (t, *J* = 2.2 Hz, 1H), 8.14 (d, *J* = 1.8 Hz, 4H); ^{13}C NMR (100 MHz, $CDCl_3$) δ 31.98, 34.76, 79.36, 82.08, 109.11, 116.42, 123.69, 123.89, 124.90, 125.21, 128.12, 138.69, 139.90, 143.51; MALDI-TOF MS (*m*/*z*): M^+ calcd for $C_{48}H_{52}N_2$, 656.41; found, 656.41. Anal. Calcd for $C_{48}H_{52}N_2$: C, 87.76; H, 7.98; N, 4.26. Found: C, 87.75; H, 7.88; N, 4.12.

*9,9',9'',9'''-(5'-((Trimethylsilyl)ethynyl)-[1,1':3',1''-terphenyl]-3,3'',5,5''-tetrayl)tetrakis(3,6-di-tert-butyl-9H-carbazole) (**6**).*

In a solvent mixture of EtOH (10 mL) and toluene (10 mL), **4** (529 mg, 1.59 mmol), **5** (2.67 g, 3.52 mmol), and $Pd(PPh_3)_4$ (127 mg, 0.110 mmol) were dissolved, and then potassium carbonate (1.27 g, 9.19 mmol) in 10 mL of H_2O was added. The reaction mixture was stirred at 90 °C for 22 h under nitrogen atmosphere. After cooling, the solvent was removed on a rotary evaporator. The residue was extracted with chloroform (200 mL) and water (100 mL) in a separation funnel. The organic layer was separated and further washed with water (100 mL) and sat. brine (100 mL). The obtained organic solution was dried over anhydrous $MgSO_4$. The solvent was removed on a rotary evaporator and the residue was purified by silica gel column chromatography using chloroform–hexane (1:8, v/v). The obtained white solid was washed with hot cyclohexane to obtain **6** as a white powder (1.19 g, 0.829 mmol, 52%), which contained a small amount of uncharacterized byproduct. Since this could readily be removed in the next step, further purification was not carried out. Owing to the contamination, a clear ^{13}C NMR spectrum was not obtained; ^{1}H NMR (400 MHz, $CDCl_3$) δ 0.24 (s, 9H), 1.44 (s, 72H), 7.45 (dd, *J* = 1.8 and 8.8 Hz, 8H), 7.49 (d, *J* = 8.8 Hz, 8H), 7.79 (t, *J* = 1.8 Hz, 2H), 7.81 (d, *J* = 1.8 Hz, 2H), 7.84 (m, 1H), 7.87 (d, *J* = 1.8 Hz, 4H), 8.12 (d, *J* = 1.8 Hz, 8H); MALDI-TOF MS (*m*/*z*): $[M + H]^+$ calcd for $C_{103}H_{114}N_4Si + H^+$, 1435.89; found, 1435.90. Anal. Calcd for $C_{103}H_{114}N_4$: C, 86.14; H, 8.00; N, 3.90. Found: C, 85.96; H, 7.67; N, 3.71.

9,9',9'',9'''-(5'-Ethynyl-[1,1':3',1''-terphenyl]-3,3'',5,5''-tetrayl)tetrakis(3,6-di-tert-butyl-9H-carbazole) (7).

Compound **6** (4.02 g, 2.80 mmol) and tetrabutylammonium fluoride (7.88 g, 30.1 mmol) were dissolved in a solvent mixture of THF (20 mL) and dichloromethane (200 mL) with cooling at 0 °C under nitrogen atmosphere. Then, the reaction mixture was stirred for 1 h at the same temperature. The solvent was removed on a rotary evaporator. The residue was dissolved in chloroform (200 mL) and then the solution was washed with water (100 mL × 2) and sat. brine (100 mL). The obtained organic solution was dried over anhydrous $MgSO_4$. The solvent was removed on a rotary evaporator and the residue was purified by silica gel column chromatography using chloroform–hexane (1:4, v/v) as an eluent. The obtained solid was washed with hot cyclohexane to obtain **7** as a white powder (2.33 g, 1.71 mmol, 61 %); ^{1}H NMR (400 MHz, $CDCl_3$) δ 1.44 (s, 72H), 3.16 (s, 1H), 7.45 (dd, *J* = 1.8 and 8.8 Hz, 8H), 7.49 (d, *J* = 8.8 Hz, 8H), 7.80 (t, *J* = 1.8 Hz, 2H), 7.84 (d, *J* = 1.8 Hz, 2H), 7.87

(d, J = 1.8 Hz, 4H), 7.88 (t, J = 1.8 Hz, 1H), 8.12 (d, J = 1.4 Hz, 8H); ^{13}C NMR (100 MHz, $CDCl_3$) δ 31.98, 34.74, 78.52, 82.79, 109.13, 116.42, 123.63, 123.66, 123.72, 123.84, 126.96, 130.57, 138.86, 140.35, 140.91, 143.25, 143.32 (one aromatic carbon signal is masked by another signal); MALDI-TOF MS (m/z): M^+ calcd for $C_{100}H_{106}N_4$, 1363.85; found, 1364.78. Anal. Cacd for $C_{100}H_{106}N_4$: C, 88.06; H, 7.83; N, 4.11. Found: C, 88.19; H, 7.93; N, 4.23.

2,7-Bis(4-hexylthiophen-2-yl)dipyrido[3,2-a:2',3'-c]phenazine.

A mixture of 2,7-dibromodipyrido[3,2-*a*:2',3'-*c*]phenazine (3.14 g, 7.14 mmol), 4-hexyl-2-(4,4,5,5-tetramethyl-1,3,2-dioxaborolan-2-yl)thiophene (4.25 g, 14.4 mmol), $Pd(PPh_3)_4$ (0.844 g, 0.73 mmol), and potassium carbonate (5.64 g, 40.8 mmol) in a solvent mixture of toluene (32 mL), ethanol (32 mL), and water (32 mL) was heated with stirring at 85 ºC for 30 h under a nitrogen atmosphere. After cooling, the solvent was removed on a rotary evaporator. The residue was dissolved in chloroform (200 mL) and then the solution was washed with water (100 mL × 2) and sat. brine (100 mL). The obtained organic solution was dried over anhydrous $MgSO_4$. The solvent was removed on a rotary evaporator and the residue was purified by silica gel column chromatography using chloroform as an eluent to obtain 2,7-bis(4-hexylthiophen-2-yl)dipyrido[3,2-*a*:2',3'-*c*]phenazine as a yellow powder (2.83 g, 4.60 mmol, 65%); ^{1}H NMR (400 MHz, $CDCl_3$) δ 0.91–0.95 (t, J = 7.3 Hz, 6H), 1.33–1.39 (m, 8H), 1.41–1.46 (m, 4H), 1.74 (quint, J = 7.3 Hz, 4H), 2.73 (t, J = 7.3 Hz, 4H), 7.09 (d, J = 1.4 Hz, 2H), 7.55 (d, J = 1.4 Hz, 2H), 7.92–7.94 (dd, J = 3.2 and 6.4 Hz, 2H), 8.39–8.41 (dd, J = 3.2 and 6.4 Hz, 2H), 9.43–9.44 (d, J = 2.8 Hz, 2H), 9.66–9.67 (d, J = 2.8 Hz, 2H), ; ^{13}C NMR (100 MHz, $CDCl_3$) δ 14.17, 22.71, 29.16, 30.52, 30.64, 31.76, 121.42, 126.30, 126.46, 128.10, 129.26, 130.07, 130.31, 139.53, 140.31, 141.89, 144.73, 145.92, 148.94; MALDI-TOF MS (m/z): M^+ calcd for $C_{38}H_{38}N_4S_2$, 614.25; found, 614.09. Anal. Calcd for $C_{38}H_{38}N_4S_2$: C, 74.23; H, 6.23; N, 9.11. Found: C, 73.97; H, 6.35; N, 8.85.

Precursor platinum(II) complex ***Pt-pre-2****.*

To 100 mL of THF, $Pt(DMSO)_2Cl_2$ (610 mg, 1.44 mmol) was added, and the mixture was refluxed for 1 h. The complex was dissolved in THF and then a solution of 2,7-bis(4-hexylthiophen-2-yl)dipyrido[3,2-*a*:2',3'-*c*]phenazine (589 mg, 0.96 mmol) in 10 mL of THF was added. The reaction mixture was heated at reflux with stirring for 19 h. After cooling, the solvent was removed on a rotary evaporator. The residue was thoroughly washed with hexane and water to give **Pt-pre-2** as a yellow powder (786 g, 0.89 mmol, 93%); ^{1}H NMR (400 MHz, $CDCl_3$) δ 0.94 (t, J = 7.3 Hz, 6H), 1.35–1.45 (m, 12H), 1.73 (quint, J = 7.3 Hz, 4H), 2.69–2.73 (t, J = 7.3 Hz, 4H), 7.18 (s, 2 H), 7.60 (s, 2H), 8.07 (dd, J = 3.2 and 6.8 Hz, 2H), 8.51 (dd, J = 3.2 and 6.8 Hz, 2H), 9.75 (d, J = 1.8 Hz, 2H), 10.1 (d, J = 1.8 Hz, 2H); MALDI-TOF MS (m/z): $[M + H]^+$ calcd for $C_{38}H_{38}Cl_2N_4PtS_2 + H^+$, 880.16; found, 880.35. Anal. Calcd for $C_{38}H_{38}Cl_2N_4PtS_2$: C, 51.82; H, 4.35; N, 6.36. Found: C, 51.94; H, 4.47; N, 6.00. The ^{13}C NMR spectrum was not obtained, due to low solubility in any solvent.

Dppz–platinum(II)–phenylacetylide complex ***Pt-1a.***

To a mixture of **Pt-pre-1** (230 mg, 0.190 mmol) and copper(I) iodide (12.0 mg, 0.0630 mmol) in di-*iso*-propylamine (2.0 mL), **3** (326 mg, 0.496 mmol) in dichloromethane (16 mL) was added. Then,

the mixture was stirred at rt for 25 h under nitrogen atmosphere. The solvent was removed on a rotary evaporator and the residue was extracted with dichloromethane (100 mL). Insoluble solids were removed by filtration and the filtrate was evaporated to dryness. The residue was purified by silica gel column chromatography using a chloroform–hexane mixture (1:2, v/v) as an eluent. Further purification was carried out by recrystallization from ethyl acetate, affording **Pt-1a** as a yellow powder (234 mg, 0.0953 mmol, 50%); ^{1}H NMR (400 MHz, CD_2Cl_2) δ 0.30–0.44 (m, 8H), 0.67 (t, *J* = 6.8 Hz, 12H), 0.79 (quint, *J* = 6.8 Hz, 8H), 0.83–0.90 (m, 8H), 0.99 (quint, *J* = 6.8 Hz, 8H), 1.44 (s, 72H), 1.55–1.64 (m, 4H), 1.72–1.79 (m, 4H), 7.13 (d, *J* = 7.3 Hz, 2H), 7.27–7.33 (m, 4H), 7.35 (t, *J* = 7.3 Hz, 2H), 7.49 (dd, *J* = 1.2 and 8.8 Hz, 8H), 7.57 (d, *J* = 7.3 Hz, 2H), 7.67 (d, *J* = 8.8 Hz, 8H), 7.72 (s, 2H), 7.95–7.99 (m, 8H), 8.10–8.12 (m, 2H), 8.18 (d, *J* = 1.4 Hz, 8H), 8.54–8.57 (m, 2H), 10.18 (d, *J* = 1.8 Hz, 2H), 10.60 (d, *J* = 1.8 Hz, 2H); ^{13}C NMR (100 MHz, $CDCl_3$) δ 14.04, 22.43, 23.53, 29.39, 31.49, 32.00, 34.70, 40.05, 55.29, 87.76, 101.59, 109.80, 116.16, 120.47, 120.84, 120.97, 121.45, 122.65, 123.59, 123.68, 125.90, 126.83, 127.86, 128.15, 129.38, 129.82, 131.02, 132.31, 132.71, 138.84, 139.25, 139.70, 139.73, 139.83, 142.83, 142.95, 143.49, 147.97, 151.25, 151.44, 152.31 (one aromatic carbon signal is masked by another signal); MALDI-TOF MS (*m/z*): $[M + H]^+$ calcd for $C_{164}H_{176}N_8{}^{195}Pt + H^+$, 2453.37; found, 2453.38. Anal. Calcd for $C_{164}H_{176}N_8Pt$: C, 80.26; H, 7.23; N, 4.57. Found: C, 80.48; H, 7.09; N, 4.76.

Dppz–platinum(II)–phenylacetylide complex ***Pt-1b.***

To a mixture of **Pt-pre-1** (200 mg, 0.165 mmol) and copper(I) iodide (10.0 mg, 0.0525 mmol) in di-*iso*-propylamine (1.8 mL), **7** (586 mg, 0.430 mmol) in dry dichloromethane (10 mL) was added, and the mixture was stirred at rt for 22 h under nitrogen atmosphere. The solvent was removed on a rotary evaporator and the residue was extracted with dichloromethane (100 mL). Insoluble solids were removed by filtration and the filtrate was evaporated to dryness. The residue was purified by silica gel column chromatography using the chloroform–hexane mixture (1:2, v/v) as an eluent. Further purification was carried out by recrystallization from ethyl acetate, affording **Pt-1b** as a yellow powder (241 mg, 0.0623 mmol, 38 %); ^{1}H NMR (400 MHz, CD_2Cl_2) δ 0.33–0.40 (m, 8H), 0.52 (t, *J* = 8.8 Hz, 12H), 0.67–0.90 (m, 24H), 1.37 (s, 144H), 1.64–1.76 (m, 8 H), 7.03–7.08 (m, 4H), 7.17 (t, *J* = 7.6 Hz, 2H), 7.37–7.38 (m, 2H), 7.40 (dd, *J* = 1.8 and 8.8 Hz, 16H), 7.49 (d, *J* = 7.6 Hz, 2H), 7.53 (d, *J* = 8.8 Hz, 16H), 7.77–7.78 (m, 6H), 7.81 (d, *J* = 1.8 Hz, 2H), 7.86 (dd, *J* = 1.8 and 7.6 Hz, 2H), 7.98 (d, *J* = 1.8 Hz, 8H), 8.09–8.10 (m, 2H), 8.08 (d, *J* = 1.8 Hz, 16H), 8.13 (d, *J* = 1.8 Hz, 4H), 8.51 (dd, *J* = 1.8 and 7.6 Hz, 2H), 10.05 (d, *J* = 1.8 Hz, 2H), 10.59 (d, *J* = 1.8 Hz, 2H); ^{13}C NMR (100 MHz, $CDCl_3$) δ 13.94, 22.29, 23.63, 29.37, 31.49, 31.95, 34.67, 40.15, 55.35, 86.63, 102.23, 109.30, 116.26, 120.30, 120.63, 121.29, 122.86, 123.02, 123.44, 123.62, 123.87, 124.05, 126.23, 126.88, 127.87, 129.22, 129.73, 129.77, 130.43, 131.40, 132.41, 133.09, 138.85, 139.61, 140.16, 142.74, 143.09, 143.34, 144.18, 147.96, 150.91, 151.50, 152.27 (three aromatic carbon signals are masked by other signals); MALDI-TOF MS (*m/z*): $[M + H]^+$ calcd for $C_{268}H_{284}N_{12}{}^{195}Pt + H^+$, 3866.22; found, 3867.00. Anal. Calcd for $C_{268}H_{284}N_{12}Pt$: C, 83.21; H, 7.40; N, 4.35. Found: C, 83.51; H, 7.38; N, 3.96.

Dppz–platinum(II)–phenylacetylide complex ***Pt-2a.***

To a mixture of **Pt-pre-2** (184 mg, 0.209 mmol) and copper(I) iodide (21.5 mg, 0.113 mmol) in di-*iso*-propylamine (2.3 mL), **3** (304 mg, 0.463 mmol) in dichloromethane (12 mL) was added, and the mixture was stirred at rt for 27 h under nitrogen atmosphere. The solvent was removed on a rotary evaporator and the residue was extracted with dichloromethane (100 mL). Insoluble solids were removed by filtration and the filtrate was evaporated to dryness. The residue was purified by silica gel column chromatography using a chloroform–hexane mixture (1:2, v/v) as an eluent. Further purification was carried out by recrystallization from ethyl acetate, affording **Pt-2a** as a yellow powder (200 mg, 0.0943 mmol, 45 %); ^{1}H NMR (400 MHz, $CDCl_3$) δ 0.88 (t, *J* = 7.1 Hz, 6H), 1.00–1.05 (m, 4H), 1.08–1.18 (m, 8H), 1.19–1.29 (m, 4H), 1.47 (s, 72H), 1.86 (t, *J* = 7.1 Hz, 4H), 5.83 (s, 2H), 6.87 (s, 4H), 7.49 (dd, *J* = 1.8 and 8.8 Hz, 8H), 7.61–7.65 (m, 12H), 7.80 (d, *J* = 1.8 Hz, 4H), 8.18 (d, *J* = 1.8 Hz, 8H), 9.45 (s, 2H), 9.86 (d, *J* = 2.3 Hz, 2H); ^{13}C NMR (100 MHz, $CDCl_3$) δ 14.20, 22.65, 28.67, 29.43, 29.83, 31.87, 32.07, 34.77, 89.66, 99.83, 109.73, 116.26, 122.42, 122.76, 123.55, 123.89, 125.98, 127.36, 129.01, 129.17, 130.32, 131.18, 131.29, 133.04, 136.98, 139.18, 140.20, 142.43, 143.03, 144.18, 147.22, 147.69 (one aromatic carbon signal is masked by another signal); MALDI-TOF MS (*m*/*z*): M^+ calcd for $C_{134}H_{140}N_8{}^{195}PtS_2$, 2120.03; found, 2120.23; Anal. Calcd for $C_{134}H_{140}N_8PtS_2$: C, 75.85; H, 6.65; N, 5.28. Found: C, 75.99; H, 6.76; N, 5.13.

Dppz–platinum(II)–phenylacetylide complex ***Pt-2b.***
To a mixture of **Pt-pre-2** (200 mg, 0.227 mmol) and copper(I) iodide (20.5 mg, 0.108 mmol) in di-*iso*-propylamine (2.5 mL), **7** (684 mg, 0.501 mmol) in dichloromethane (14 mL) was added, and the mixture was stirred at rt for 24 h under nitrogen atmosphere. The solvent was removed on a rotary evaporator and the residue was extracted with dichloromethane. Insoluble solids were removed by filtration and the filtrate was evaporated to dryness. The residue was purified by silica gel column chromatography using a chloroform–hexane mixture (1:2, v/v) as an eluent. Further purification was carried out by recrystallization from ethyl acetate, affording **Pt-2b** as a brownish yellow powder (378.9 mg, 0.107 mmol, 47 %); ^{1}H NMR (400 MHz, $CDCl_3$) δ 0.66 (t, *J* = 7.8 Hz, 6H), 0.94–1.02 (s, 12H), 1.08–1.12 (m, 4H), 1.35 (s, 144H), 2.00 (t, *J* = 7.8 Hz, 4H), 6.09 (s, 2H), 7.09 (s, 2H), 7.34 (dd, *J* = 1.8 and 8.8 Hz, 16H), 7.52 (d, *J* = 8.8 Hz, 16H), 7.76 (m, 2H), 7.79 (t, *J* = 1.8 Hz, 4H), 8.01 (d, *J* = 1.8 Hz, 8H), 8.03–8.10 (m, 20H), 8.12–8.15 (m, 2H), 9.85 (d, *J* = 1.8 Hz, 2H), 9.97 (s, 2H); ^{13}C NMR (100 MHz, $CDCl_3$) δ 14.12, 22.65, 28.78, 29.89, 30.05, 31.42, 31.90, 34.64, 88.00, 101.11, 109.25, 116.29, 122.86, 123.31, 123.67, 123.85, 127.46, 129.22, 129.36, 129.83, 130.33, 130.47, 131.51, 133.44, 137.12, 138.80, 140.04, 140.24, 142.59, 143.21, 144.31, 144.63, 147.74, 148.27 (three aromatic carbon signals are masked by other signals); MALDI-TOF MS (*m*/*z*): M^+ calcd for $C_{238}H_{248}N_{12}{}^{195}PtS_2$, 3532.88; found, 3532.86. Anal. Calcd for $C_{238}H_{248}N_{12}PtS_2$: C, 75.85; H, 6.65; N, 5.28. Found: C, 75.99; H, 6.76; N, 5.13.

Dppz–platinum(II)–phenylacetylide complex ***Pt-2c.***
To a mixture of **Pt-pre-2** (300 mg, 0.341 mmol) and copper(I) iodide (55.5 mg, 0.291 mmol) in di-*iso*-propylamine (3.7 mL), phenylacetylene (820 mg, 0.75 mmol) in toluene (20 mL) was added. Then, the mixture was reacted at 70 °C for 24 h under nitrogen. After the reaction, the solvent was removed

on a rotary evaporator. The obtained mixture was thoroughly washed with chloroform to give **Pt-2c** as a reddish orange powder (173 mg, 0.171 mmol, 50%). Further purification was not carried out, due to the quite low solubility in any solvents; MALDI-TOF MS (*m*/*z*): M^+ calcd for $C_{54}H_{48}N_4{}^{195}PtS_2$, 1011.30; found, 1011.12; Anal. Calcd for $C_{54}H_{48}N_4PtS_2$: C, 64.08; H, 4.78; N, 5.54. Found: C, 64.29; H, 4.57; N, 5.47.

References

S1. Okamura, N.; Egawa, K.; Maeda, T.; Yagi, S. Control of Excimer Phosphorescence by Steric Effects in Cyclometalated Platinum(II) Diketonate Complexes Bearing Peripheral Carbazole Moieties towards Application in Non-Doped White OLEDs. *New J. Chem.* **2018**, *42*, 11583–11592.

S2. Lim, Y.-K.; Jiang, X.; Bollinger, J.C.; Lee, D. Molecular Engineering of Two-Dimensional π-Conjugation: Expected and Unexpected Photophysical Consequences of a Simple Particle-in-a-Box Approach. *J. Mater. Chem.* **2007**, *17*, 1969–1980.

S3. Nguyen, N.T.; Hofkens, J.; Scheblykin, I.G.; Kruk, M.; Dehaen, W. Click Reaction Synthesis and Photophysical Studies of Dendritic Metalloporphyrins. *Eur. J. Org. Chem.* **2014**, 1766–1777.

2. Device Working Mechanisms

2.1. Single-Layer-Type Device

The schematic illustration of the working mechanism of the single-layer-type devices (S-1 and S-2) is shown in Figure S1. Holes are injected into the PEDOT:PSS hole-injection layer from the anode and then move smoothly to the thin film of the platinum(II) complex (**Pt-1** or **Pt-2**) as the emitting layer (EML) with the assistance of the hole-transporting acetylide ligands (path A in Figure S1). On the other hand, relatively small numbers of electrons should be injected from the cathode because of the low-electron-transporting ability of the platinum(II) complex (path B in Figure S1). As a result, some part of the injected holes should meet together with the electrons in the EML (path C in Figure S1), while the other part should go through the EML and be subjected to charge recombination in the CsF or Al layer (path D in Figure S1). Thus, combined with the low photoluminescence quantum yields of the platinum(II) complexes, a low probability of charge recombination in the EML seriously reduces the charge carrier balance γ to afford low device performance.

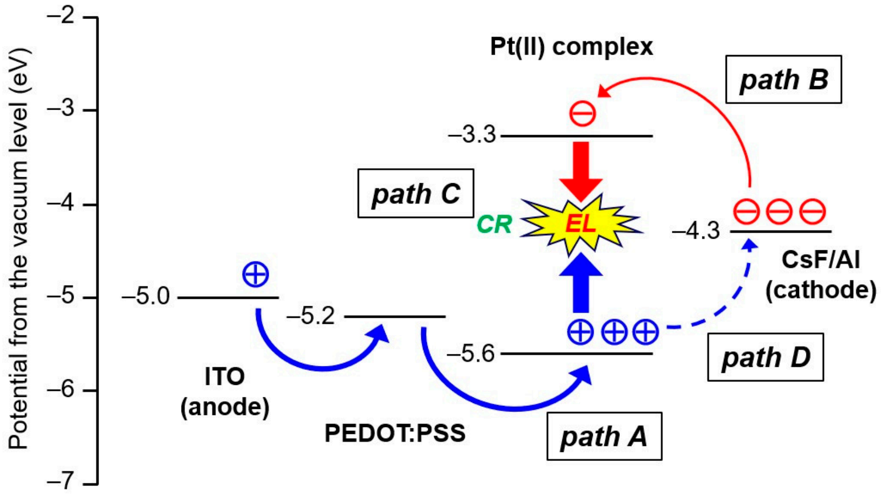

Figure S1. Schematic illustration for device working mechanism of the present single-layer-type OLED. The abbreviation CR represents charge recombination.

2.2. Multilayer Device

As shown in Figure S2, the improved device performance of devices M-1 and M-2 was achieved through the efficient injection of holes and electrons into the EML and their confinement therein. With the assistance of hole- and electron-transporting layers (PVCz and TPBi, respectively), holes and electrons are smoothly injected into the EML in energetically favorable manners (path A and path B in Figure S2, respectively). The HOMO level of the platinum(II) complex is energetically higher than that of TPBi and its LUMO level is lower than that of PVCz. Thus, the holes and electrons injected into the EML are blocked at the EML/TPBi and PVCz/EML interfaces, respectively (path C and path D in Figure S2, respectively), and confined within the EML to achieve efficient charge

recombination (path E in Figure S2). This charge carrier confinement allows us to obtain the improved charge carrier balance γ, affording better device performance in comparison with S-1 and S2.

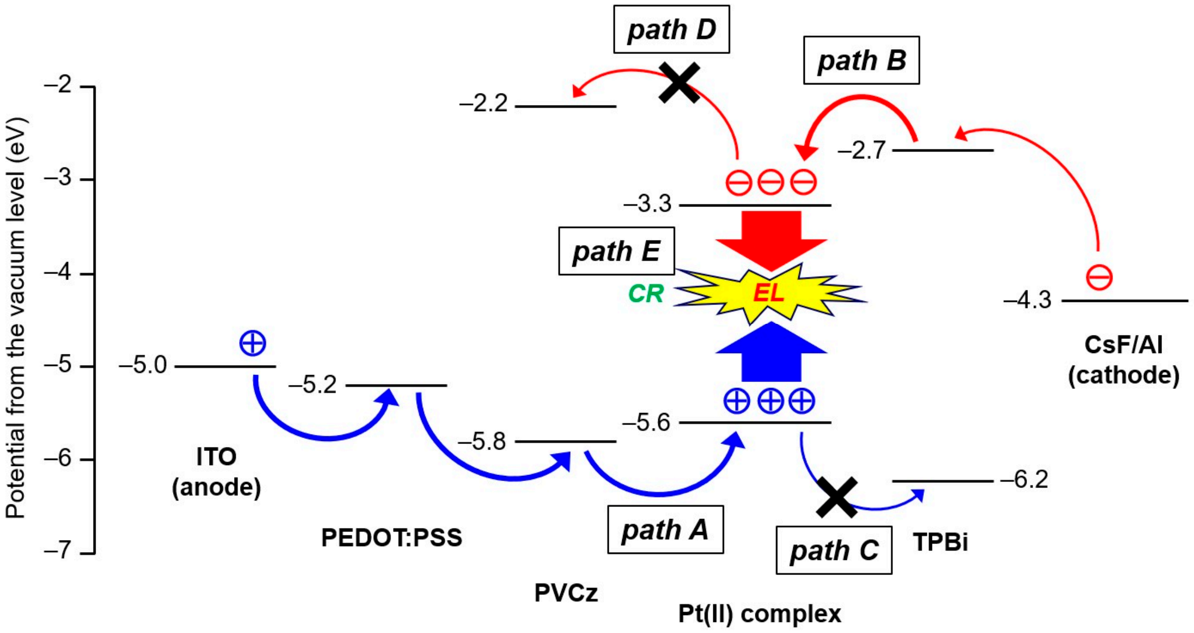

Figure S2. Schematic illustration for device working mechanism of the present multilayer OLED. The abbreviation CR represents charge recombination.

3. ^{1}H and ^{13}C NMR Spectra

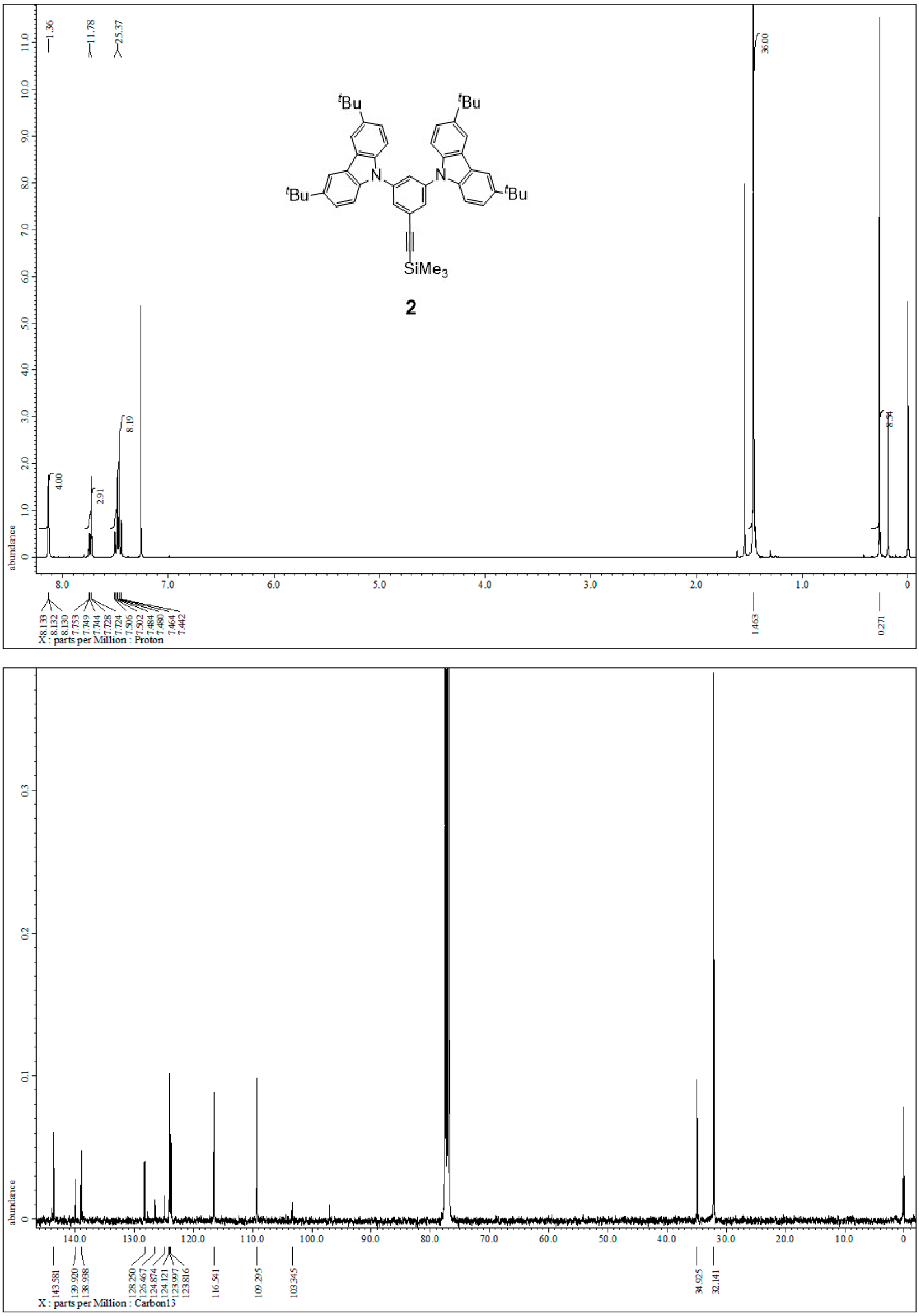

Figure S3. ^{1}H (upper, 400 MHz, $CDCl_3$) and ^{13}C (lower, 100 MHz, $CDCl_3$) NMR spectra of **2**.

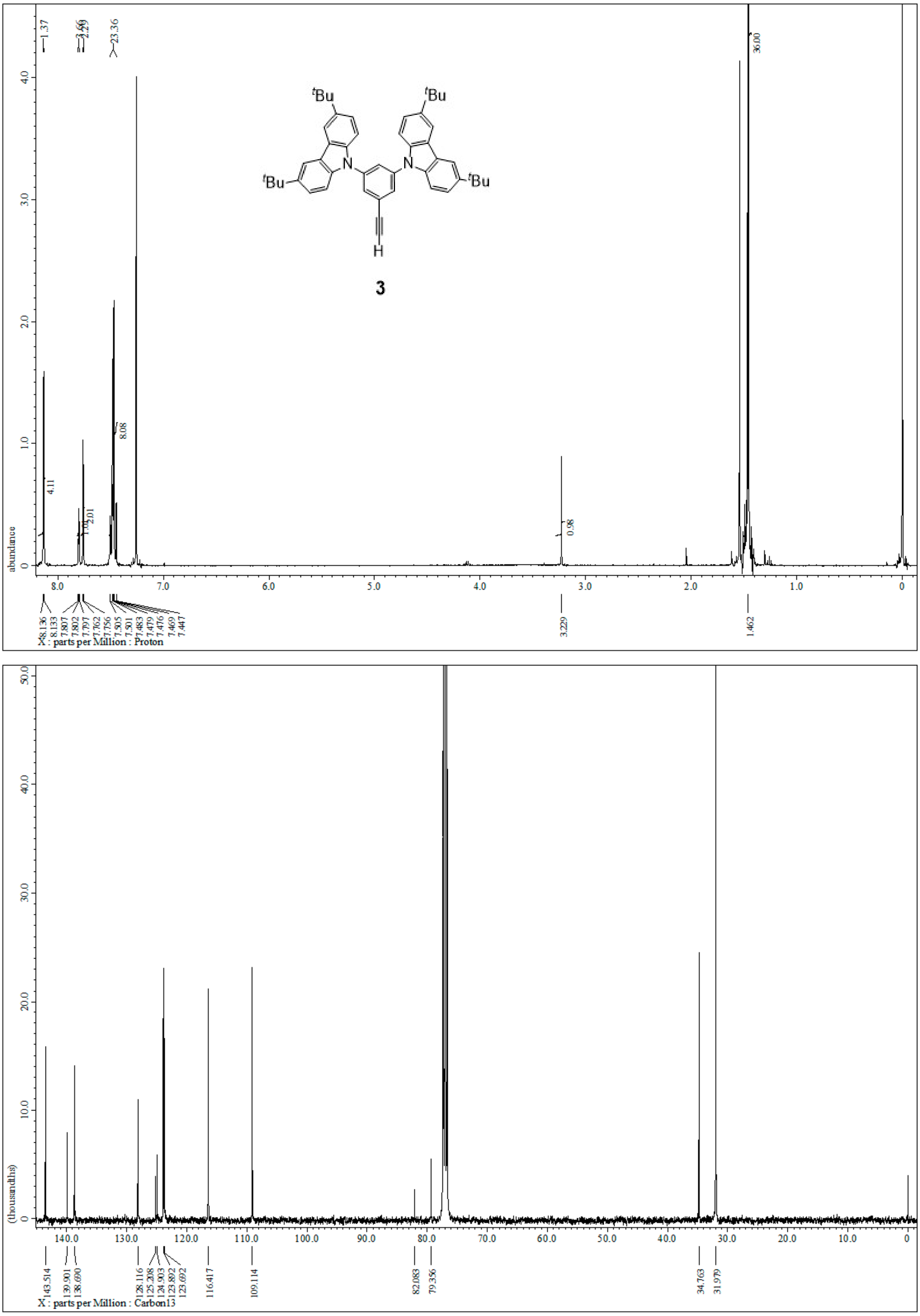

Figure S4. ^{1}H (upper, 400 MHz, $CDCl_3$) and ^{13}C (lower, 100 MHz, $CDCl_3$) spectra of **3**.

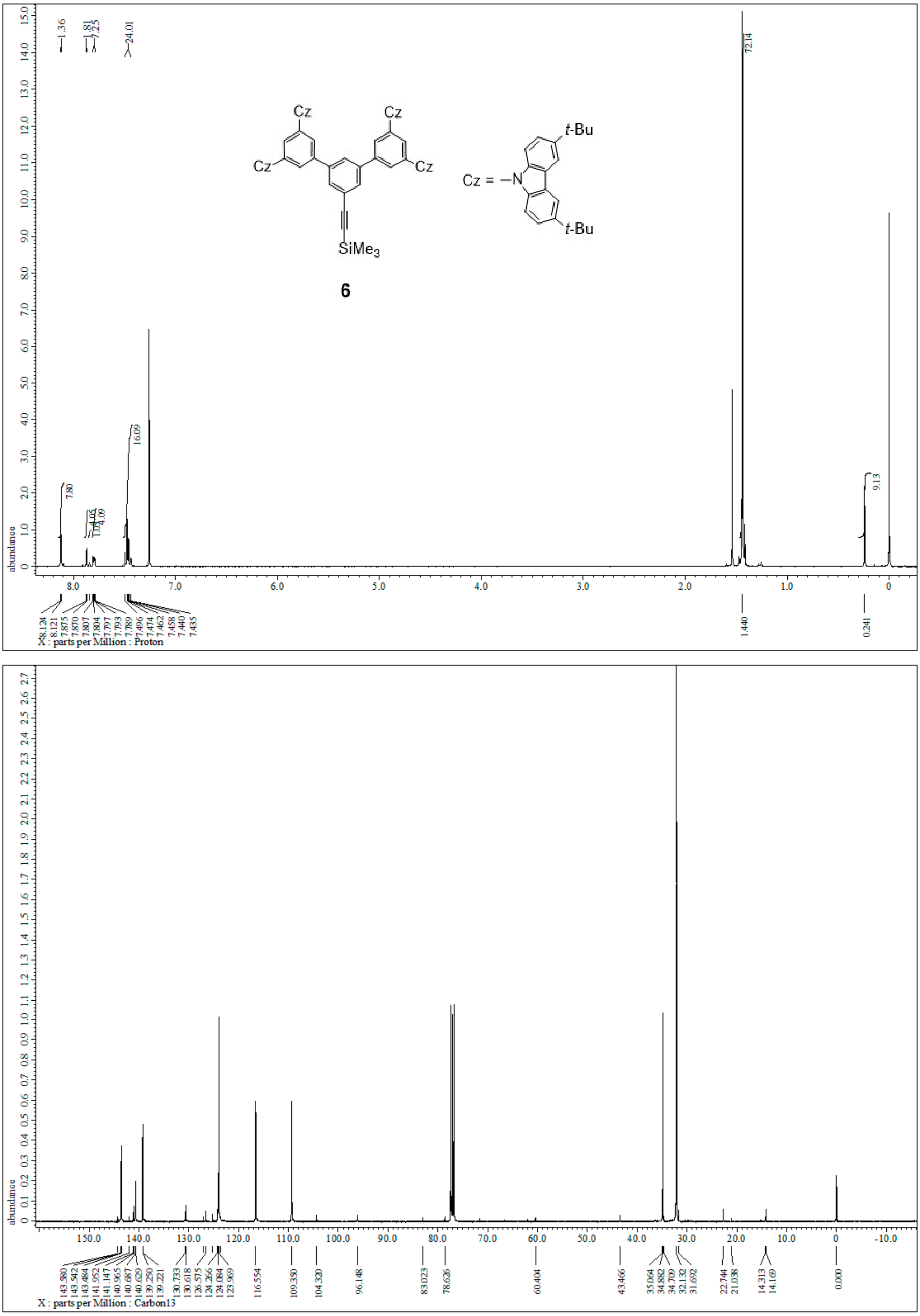

Figure S5. ^{1}H (upper, 400 MHz, $CDCl_3$) and ^{13}C (lower, 100 MHz, $CDCl_3$) NMR spectra of **6**.

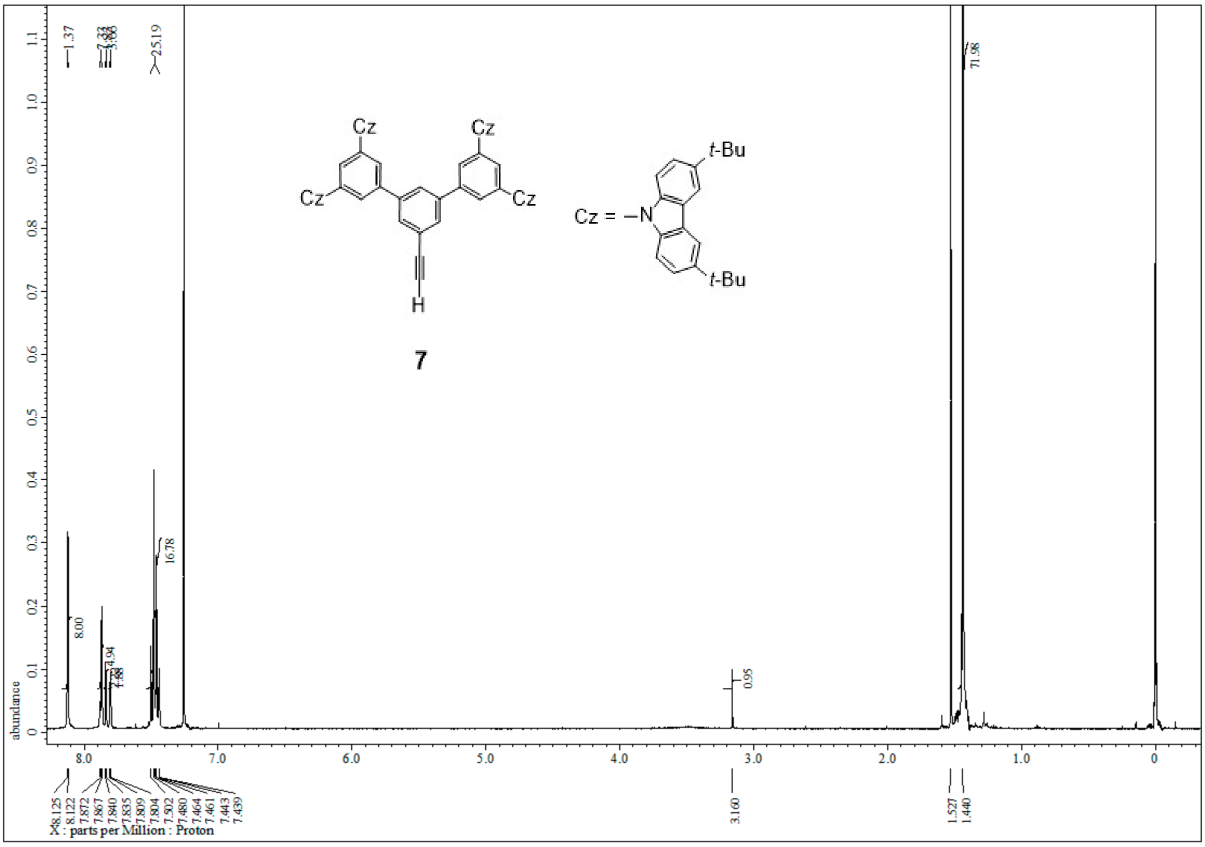

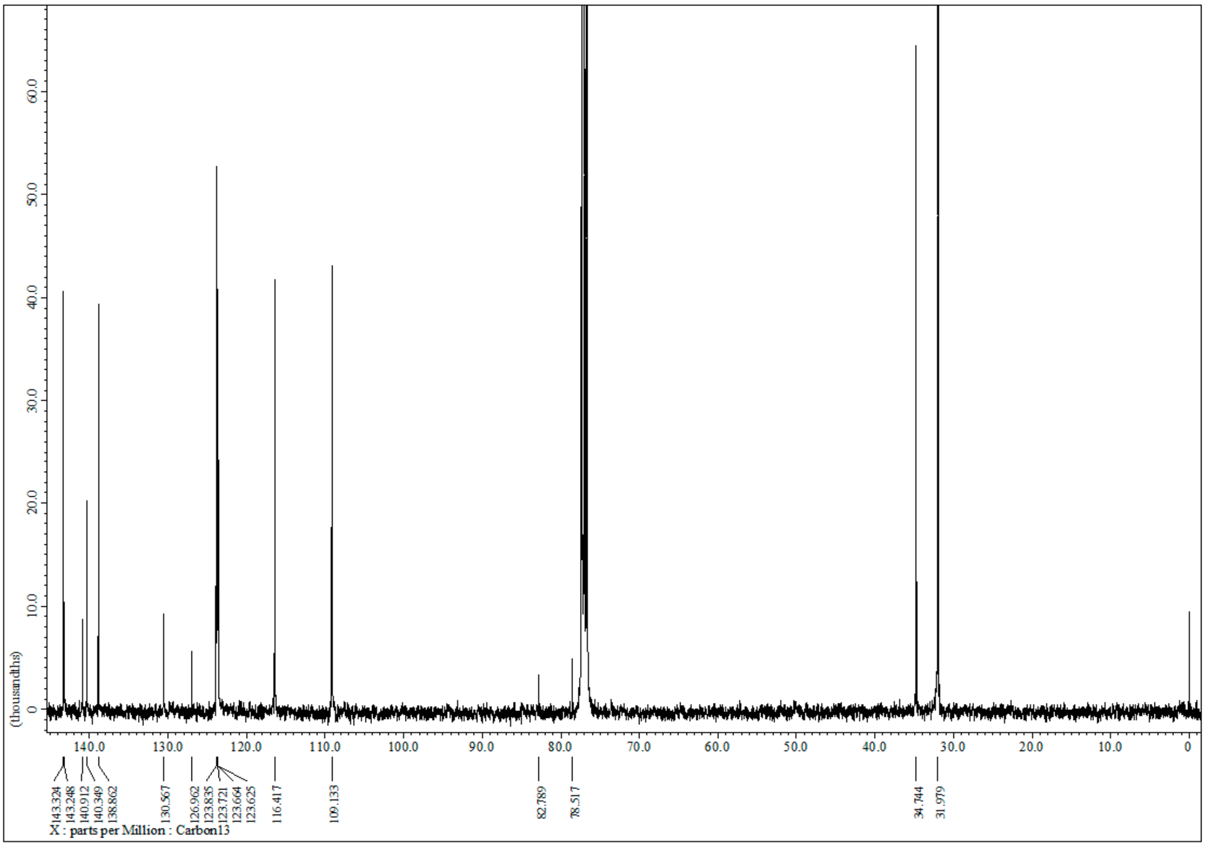

Figure S6. ^{1}H (upper, 400 MHz, $CDCl_3$) and ^{13}C (lower, 100 MHz, $CDCl_3$) NMR spectra of **7**.

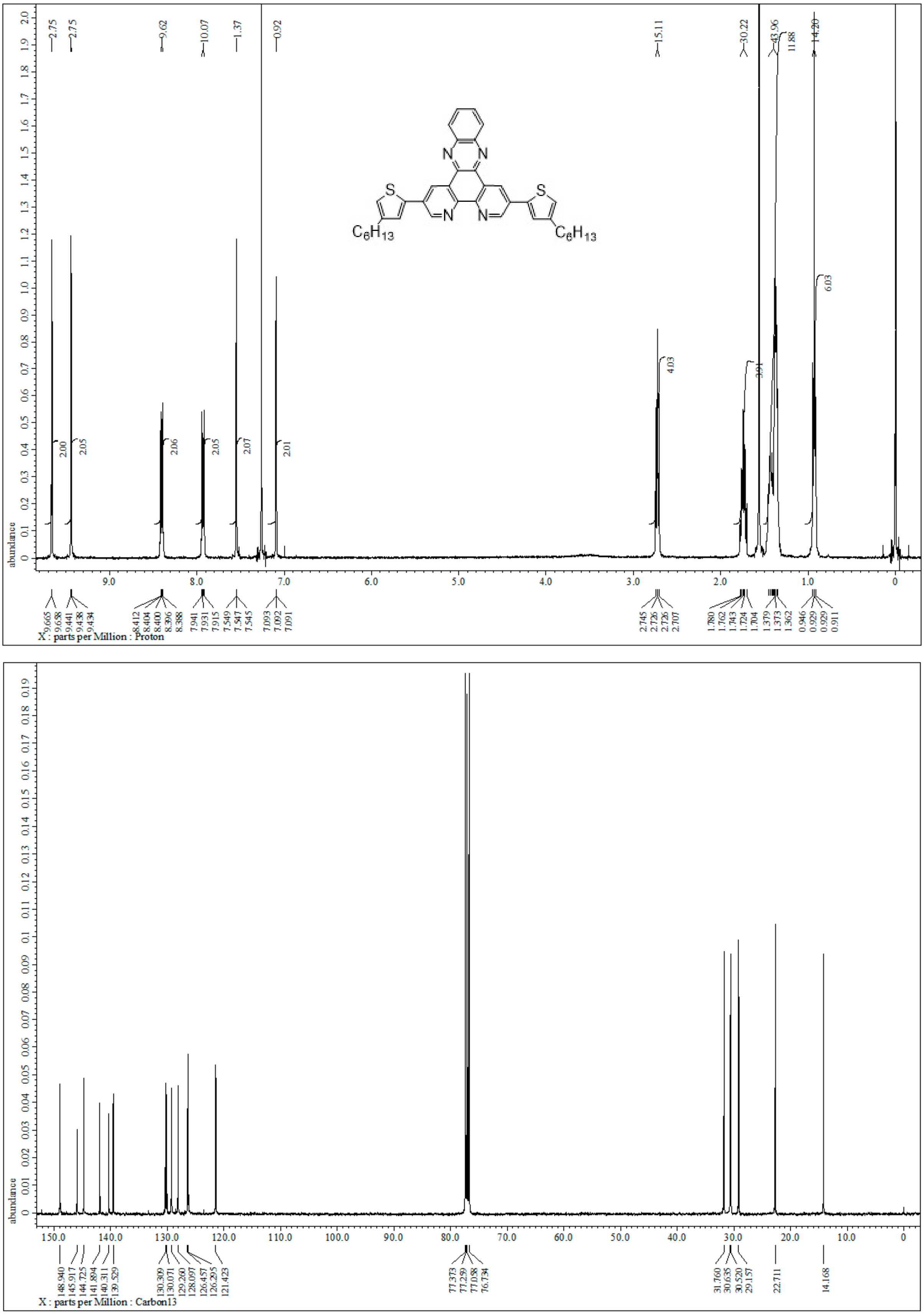

Figure S7. ^{1}H (upper, 400 MHz, $CDCl_3$) and ^{13}C (lower, 100 MHz, $CDCl_3$) NMR spectra of 2,7-bis(4-hexylthiophen-2-yl)dipyrido[3,2-*a*:2',3'-*c*]phenazine.

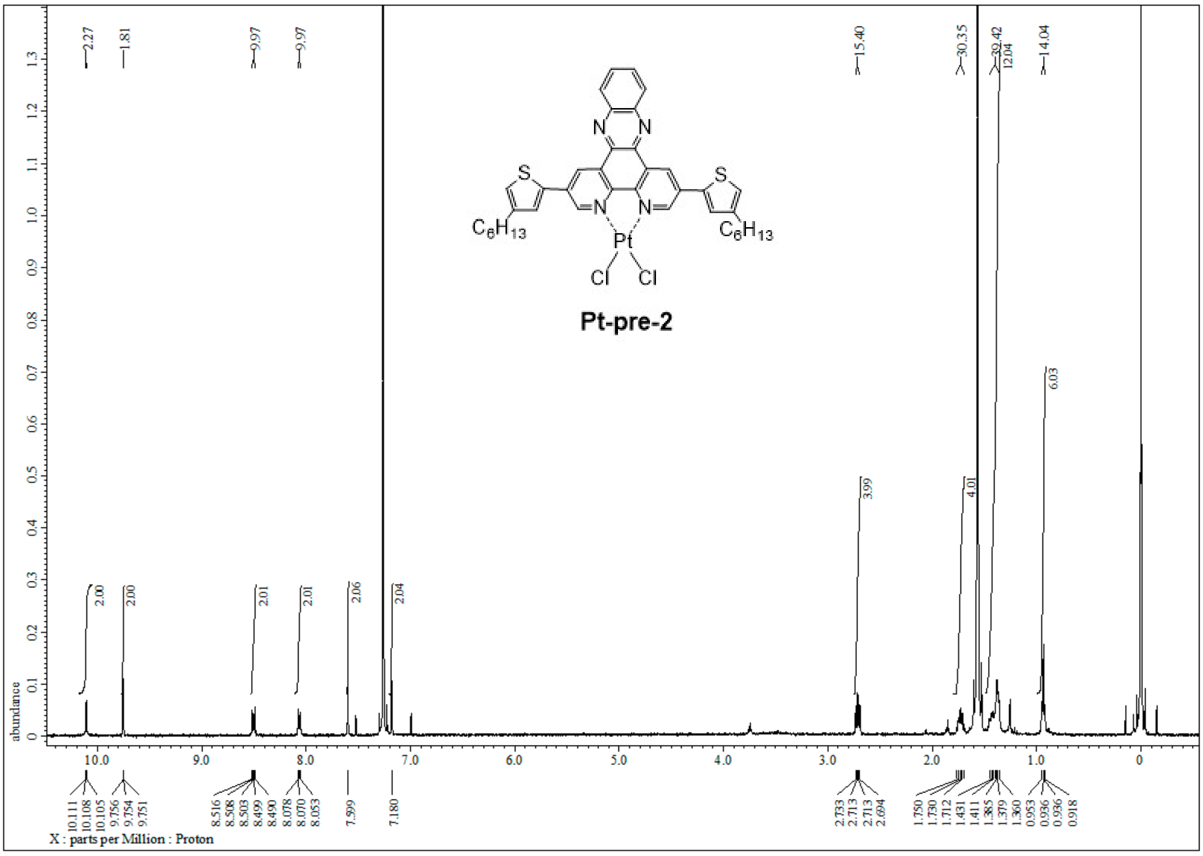

Figure S8. ^{1}H NMR spectrum (400 MHz, $CDCl_3$) of **Pt-pre-2**.

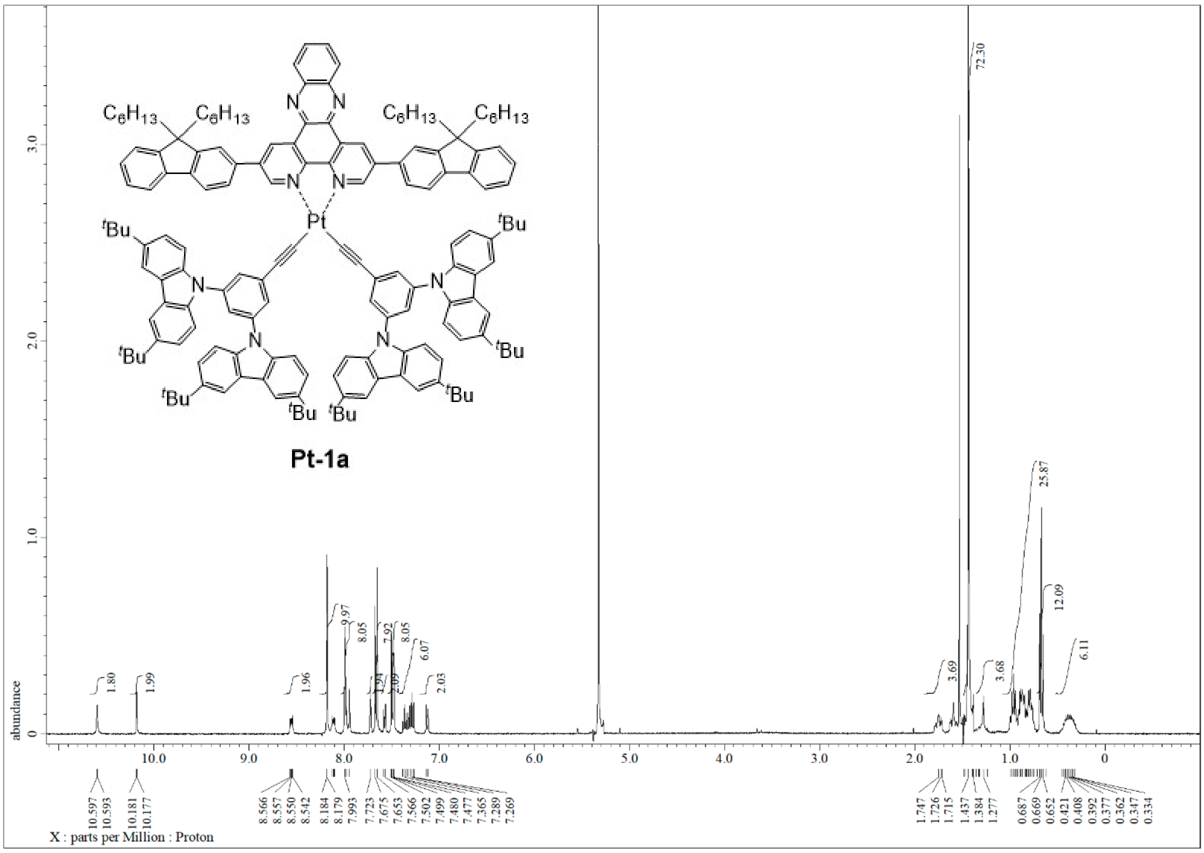

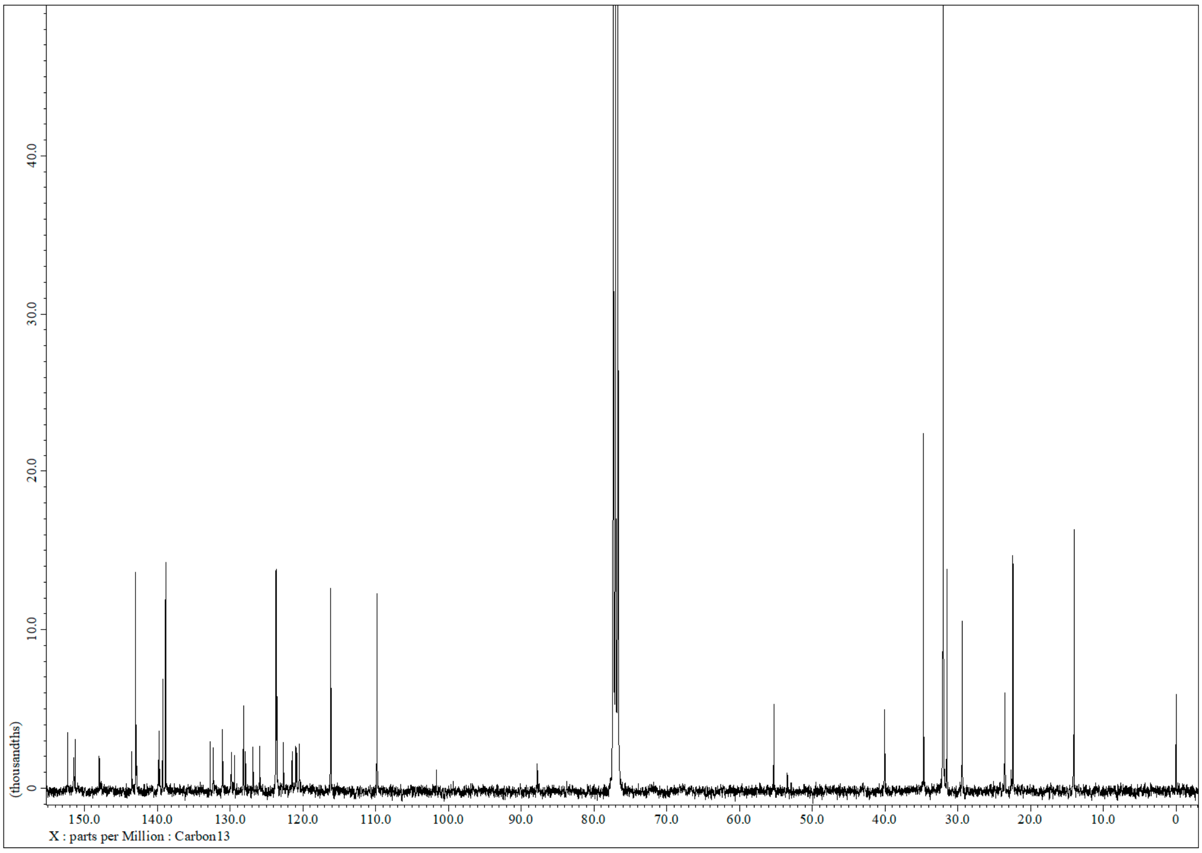

Figure S9. ^{1}H (upper, 400 MHz, CD_2Cl_2) and ^{13}C (lower, 100 MHz, $CDCl_3$) NMR spectra of **Pt-1a**.

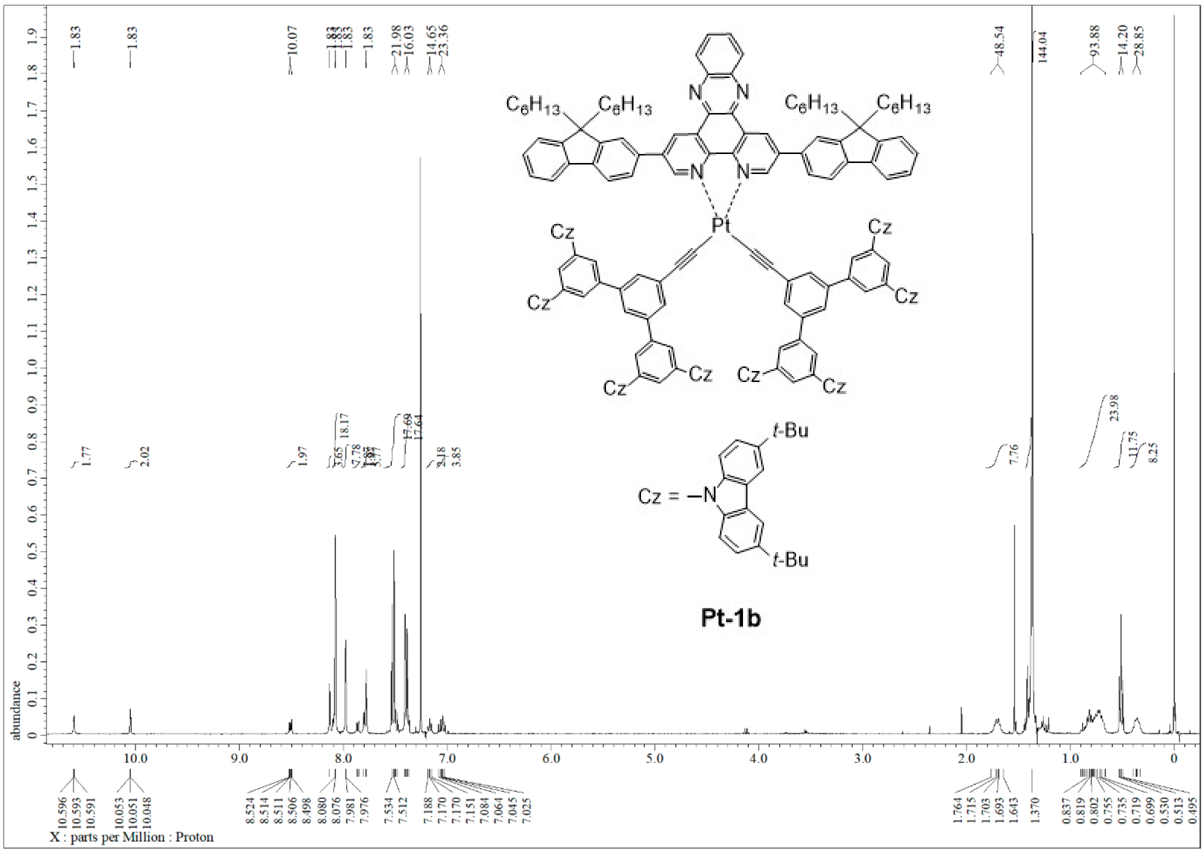

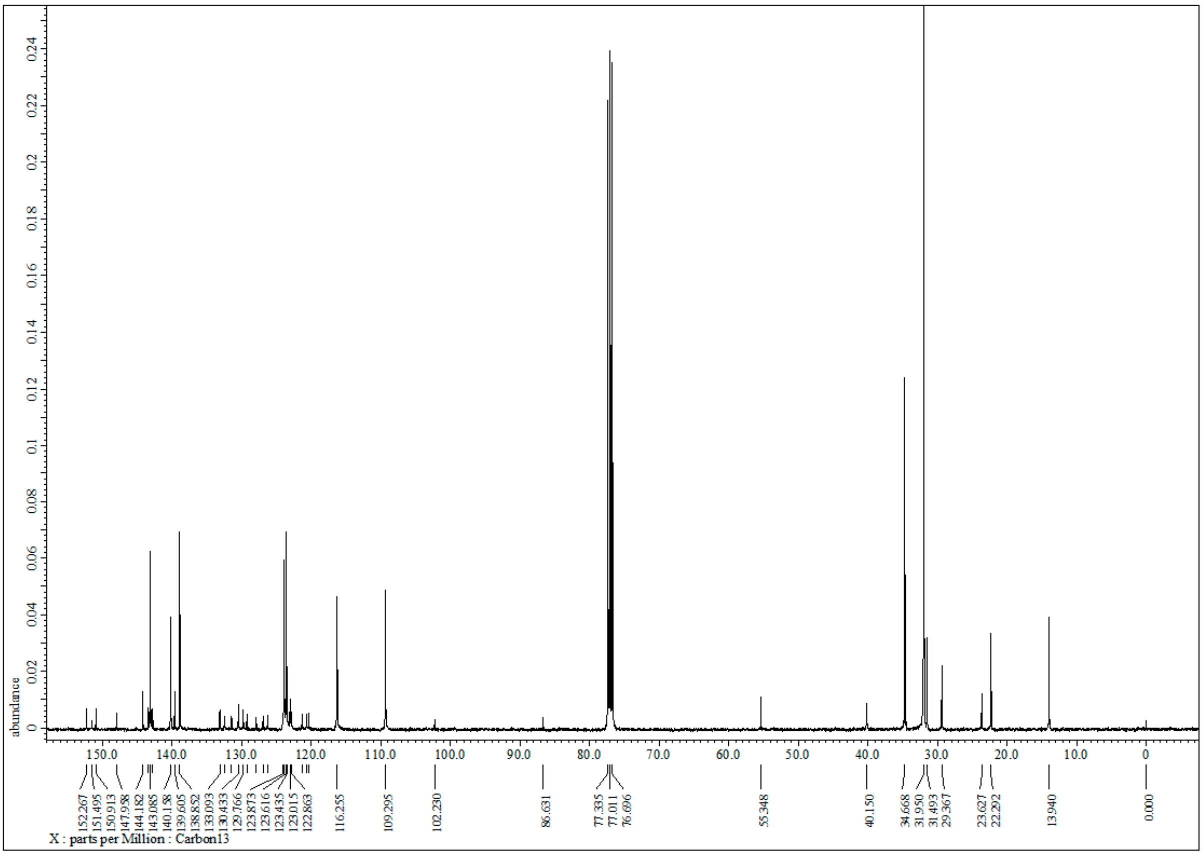

Figure S10. ^{1}H (upper, 400 MHz, CD_2Cl_2) and ^{13}C (lower, 100 MHz, $CDCl_3$) NMR spectra of **Pt-1b**.

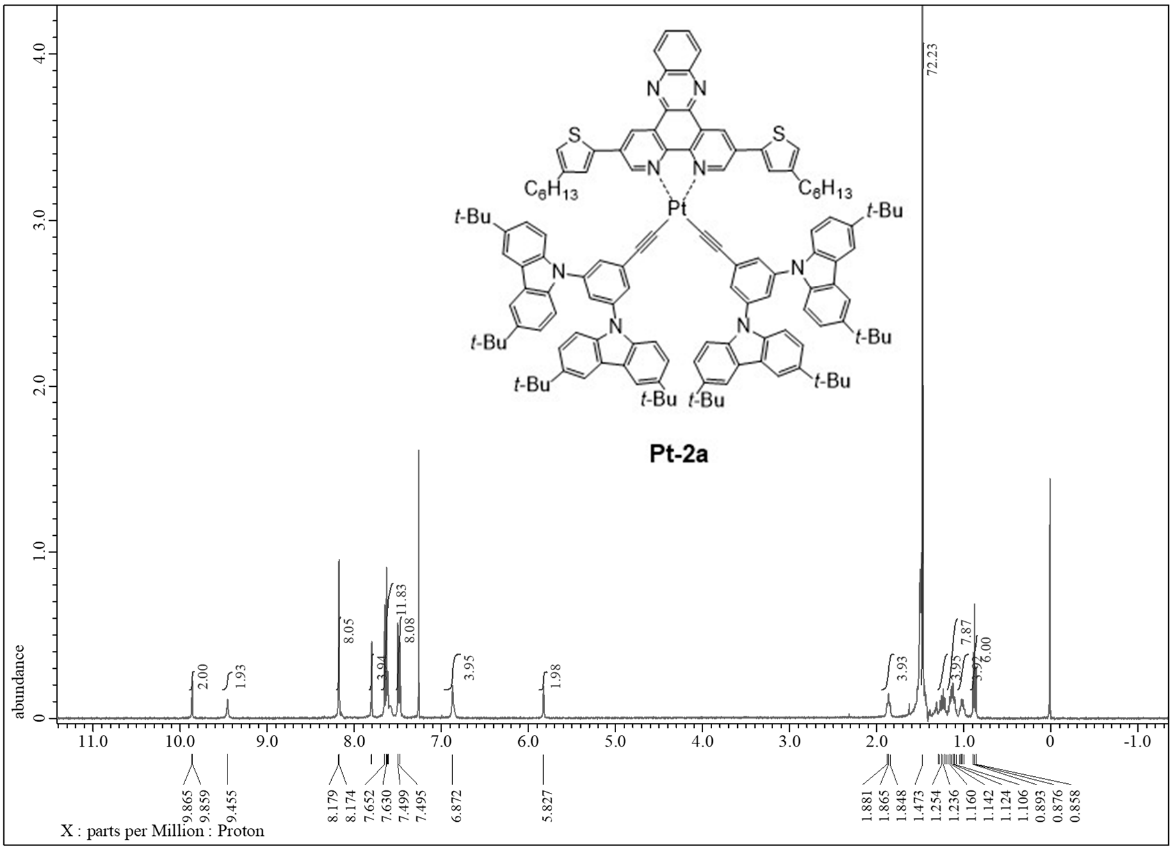

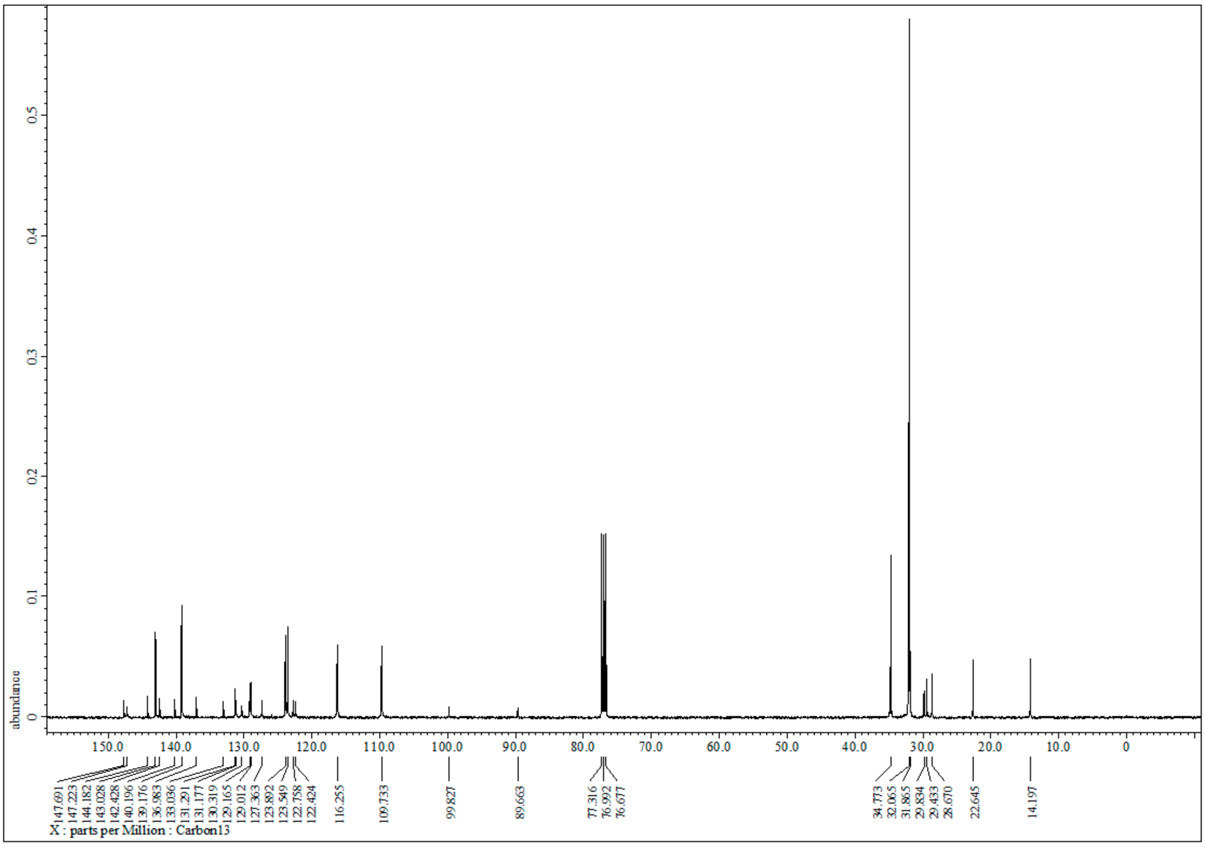

Figure S11. ^{1}H (upper, 400 MHz, CD_2Cl_2) and ^{13}C (lower, 100 MHz, $CDCl_3$) NMR spectra of **Pt-2a**.

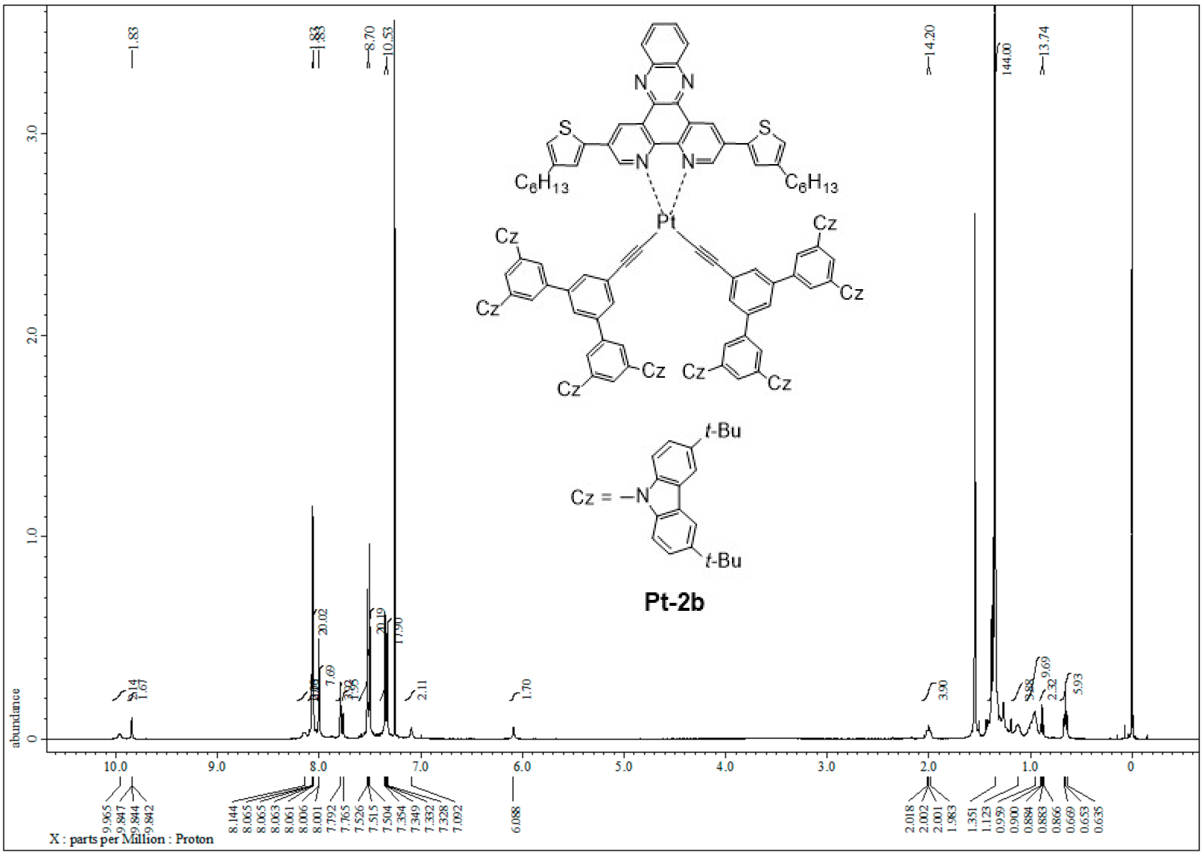

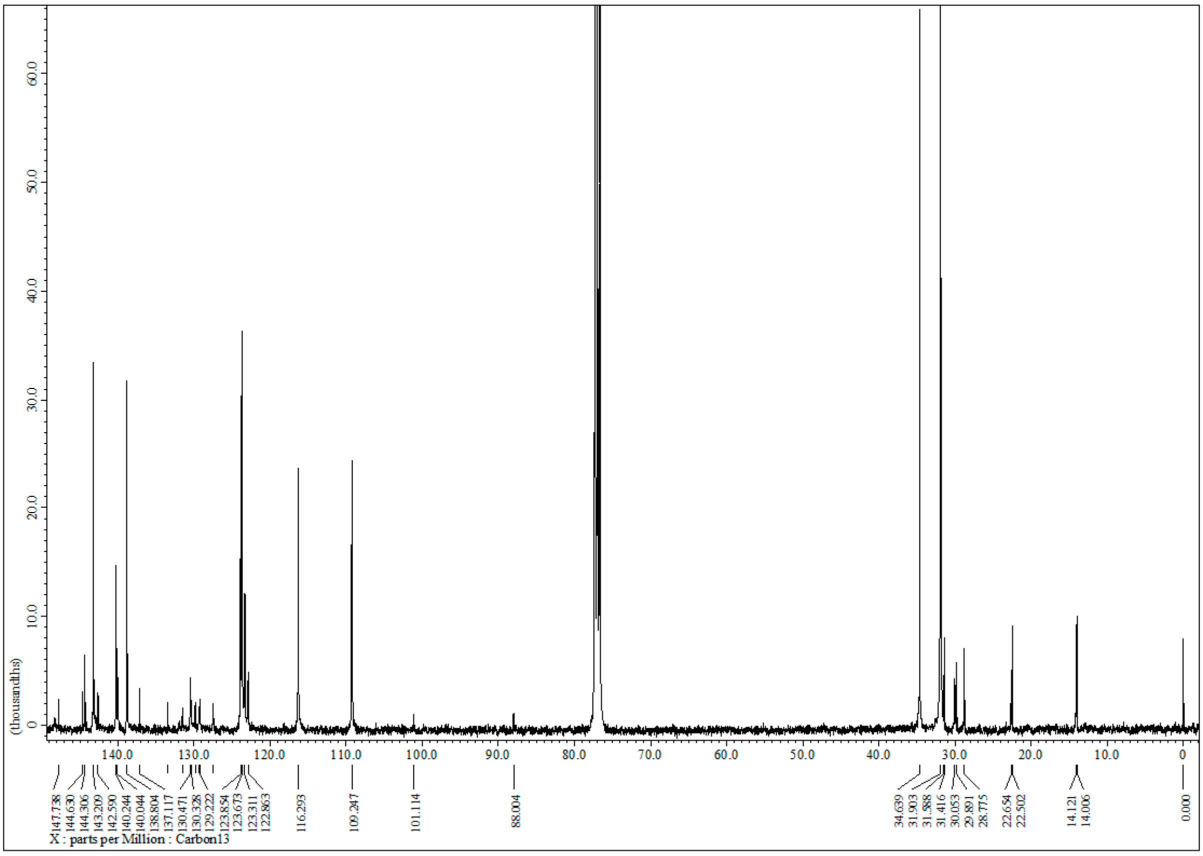

Figure S12. ^{1}H (upper, 400 MHz, CD_2Cl_2) and ^{13}C (lower, 100 MHz, $CDCl_3$) NMR spectra of **Pt-2b**.

4. Natural Transition Orbital (NTO) Analyses

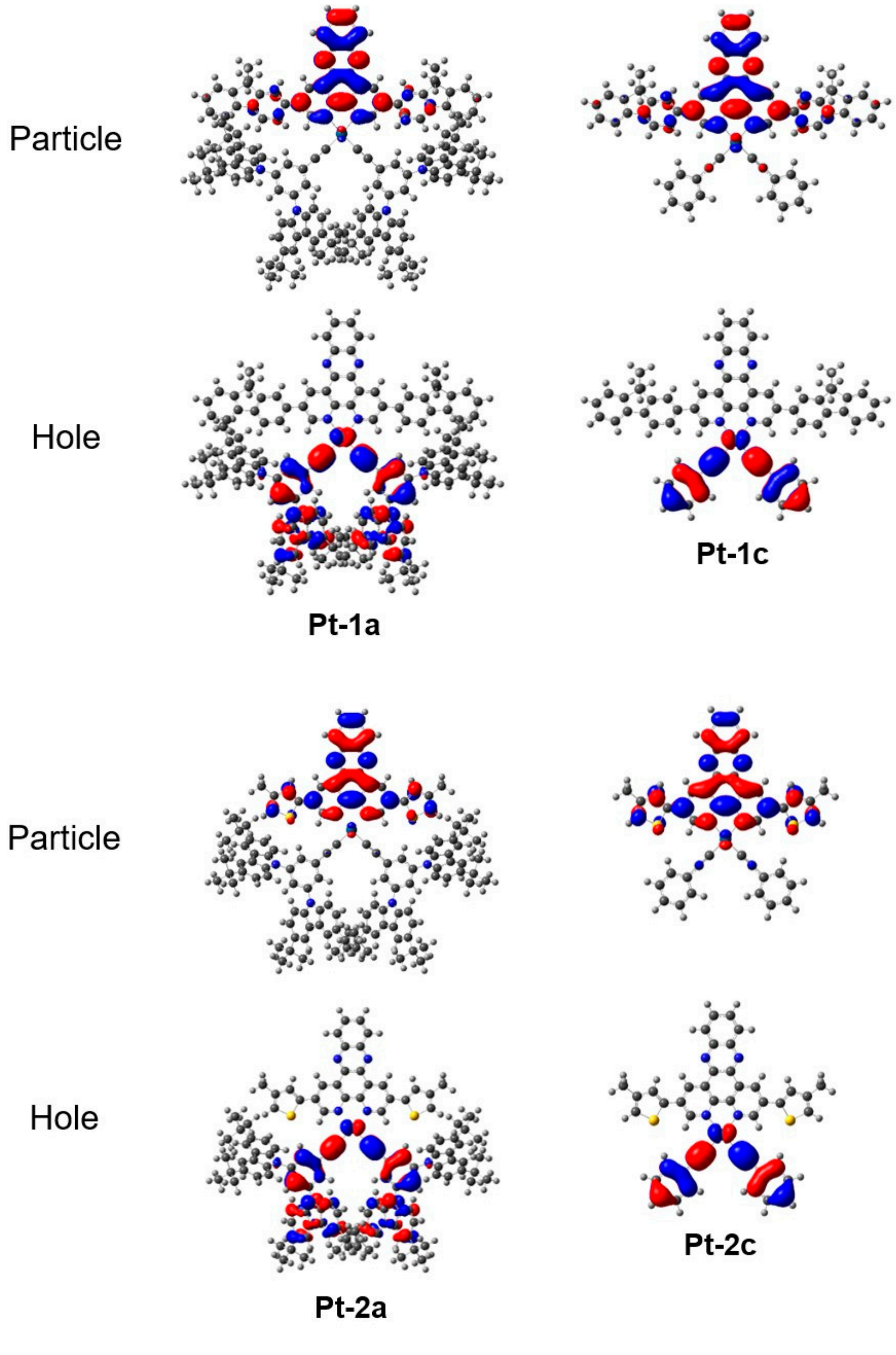

Figure S13. NTO analyses of **Pt-1a**, **Pt-1c**, **Pt-2a**, and **Pt-2c** at the T_1 geometry based on density functional theory (DFT) and time-dependent DFT (TD-DFT) calculations at the B3LYP/6-31G(d) (C, H, N, and S)/LANL2DZ (Pt) level of theory.
